# Comprehensive disproportionality analysis of individual case safety reports associated with Janus kinase inhibitors in psoriasis and psoriatic arthritis using the FAERS database

**DOI:** 10.3389/fimmu.2025.1629886

**Published:** 2025-10-02

**Authors:** Kunhong Deng, Han Jiang, Shan Xie, Minghui Yin, Chengjun Guo, Longjian Huang, Junlong Ma, Yun Kuang, Yuxia Xiang, Chengxian Guo

**Affiliations:** ^1^ Center of Clinical Pharmacology, The Third Xiangya Hospital, Central South University, Changsha, Hunan, China; ^2^ Department of Radiology, The Third Xiangya Hospital, Central South University, Changsha, Hunan, China; ^3^ School of Applied Mathematics, Guangdong University of Technology, Guangzhou, Guangdong, China; ^4^ School of Clinical Medicine, Youjiang Medical University for Nationalities, Baise, Guangxi, China

**Keywords:** Janus kinase inhibitors, psoriasis, psoriatic arthritis, FAERS, pharmacovigilance, disproportionality analysis

## Abstract

**Introduction:**

The development of Janus kinase (JAK) inhibitors has significantly expanded the therapeutic options for patients with psoriasis and psoriatic arthritis (PsA). However, the distinct pharmacological profiles and target selectivity of these agents result in varying safety implications. This study systematically evaluates the safety of different JAK inhibitors in psoriasis and PsA patients.

**Methods:**

A retrospective pharmacovigilance study was conducted using the Food and Drug Administration Adverse Event Reporting System (FAERS) database. The disproportionality analysis methods, including reporting odds ratio (ROR) and information component (IC), were used to evaluate the adverse events (AEs) associated with the use of JAK inhibitors (deucravacitinib, upadacitinib, tofacitinib) in patients with psoriasis and PsA. To reduce potential confounding factors, sensitivity analysis was carried out.

**Results:**

A total of 167,807 worldwide individual case safety reports (ICSRs) of JAK inhibitors (Q4-2014 to Q3-2024) from 10,616 psoriasis and PsA patients were identified. Skin and subcutaneous tissue disorders, infections and infestations, and gastrointestinal disorders were frequently reported AE signals for JAK inhibitors. Musculoskeletal and connective tissue disorders were prominent AEs associated with upadacitinib and tofacitinib. The reporting rates of skin and subcutaneous tissue disorder AEs for deucravacitinib were higher than those for the other two drugs, whereas most other AE reporting rates for deucravacitinib were lower. Some AEs that have not been reported in the drug prescribing information deserve further attention. Subgroup analysis suggested that female subjects had a higher likelihood of developing skin and subcutaneous tissue disorders after taking tofacitinib. Comparisons between psoriasis and PsA indicated that AE signals were generally comparable across the two indications.

**Conclusions:**

This research offers practical evidence for assessing the safety of JAK inhibitors used in psoriasis and PsA. Since disproportionality analysis serves as a hypothesis-generating approach, the results necessitate further validation in studies with denominator data to assess causal relationships.

## 1 Introduction

Psoriasis, a common chronic skin disease affecting approximately 2% of the global population, is characterized by elevated levels of pro-inflammatory cytokines (1–3). In addition to skin lesions, 30% of psoriasis patients have joint lesions and will progress to psoriatic arthritis (PsA) (4, 5). PsA is an inflammatory joint disease associated with psoriasis, which can cause symptoms such as joint pain, swelling, stiffness, and limited mobility (6, 7).Current management of psoriasis encompasses topical agents, biologic therapies, and phototherapy. Targeted therapies have gained widespread adoption due to their high selectivity, rapid onset, and favorable safety profiles (8). The Janus kinase/signal transducer and activator of transcription (JAK-STAT) pathway is an important signaling pathway involved in the pathogenesis of psoriasis and PsA. This pathway transmits extracellular signals to the cell nucleus through transmembrane receptors and mediates a series of physiological and pathological processes (9, 10).

The JAK family consists of four members including JAK1, JAK2, JAK3, and tyrosine kinase 2 (TYK2). By inhibiting the cytokine pathway, JAK inhibitors can effectively reduce the expression of key cytokines involved in the development of psoriasis and PsA (11–13). Currently, several JAK inhibitors have been approved for the treatment of psoriasis and PsA (14).Tofacitinib (approved by the FDA for PsA in 2017, a JAK1/JAK3 inhibitor) (15) and upadacitinib (approved by the FDA for PsA in 2021, a selective JAK1 inhibitor) can regulate the immune signaling pathway, and they would not cause renal and hepatic toxicity like cyclosporine A and methotrexate (16). Deucravacitinib, a TYK2 inhibitor, was approved by the FDA for moderate-to-severe plaque psoriasis in 2022 (17), which significantly improves skin clearance and disease severity (18).

JAK inhibitors used for psoriasis and PsA are associated with characteristic adverse events (AEs), including headache, diarrhea, upper respiratory infections, myelosuppression, and rash (16). With the continuous promotion and application of these drugs, some AEs may remain newly identified or underreported. Currently, there is no systematic report on safety signals of JAK inhibitors used in psoriasis and PsA. Due to the differences in the inhibitory targets and molecular activities of different JAK inhibitors, it is necessary to comprehensively investigate the safety profiles of individual drugs and conduct comparisons among patients with psoriasis and PsA.

Disproportionality analysis is a widely used method for establishing hypotheses about correlations between specified drugs and AEs, which involves signal detection through the review of individual case safety reports (ICSRs) and statistical analysis. The Food and Drug Administration Adverse Event Reporting System (FAERS) is the largest post-market surveillance program, which facilitates the early detection of AEs and the monitoring of newly launched drugs. It has been widely used to identify risk signals of AEs.

Achieving comprehensive disease control for psoriasis often requires therapies that can effectively manage both skin and joint symptoms simultaneously (16). Psoriasis and PsA share a common T-cell-mediated immunopathogenic mechanism (19), and a substantial proportion of patients with psoriasis eventually develop PsA (20), resulting in an overlap of these two conditions in clinical practice. Although the FDA has only approved the application of three JAK inhibitors for either psoriasis or PsA as one of their indications, off-label use targeting the other indication (psoriasis or PsA) has been observed in both clinical trials and clinical practice. Currently, existing clinical trials have confirmed the efficacy and safety of deucravacitinib in PsA, suggesting that it may become the first orally administered TYK2 inhibitor approved for PsA (21). Studies have found that tofacitinib has demonstrated positive effects on the treatment of chronic plaque psoriasis (22, 23), and it can serve as a therapeutic option for patients with psoriasis, with generally good tolerability (24). Furthermore, tofacitinib has been approved for the treatment of moderate to severe plaque psoriasis in Russia (25). Moreover, there have been reports of upadacitinib being used for the treatment of psoriasis (26–29). Importantly, JAK inhibitors have shown efficacy in clinical trials for both psoriasis and PsA (24, 30). In addition, several FAERS-based pharmacovigilance studies have analyzed psoriasis and PsA as a combined cohort when evaluating safety outcomes (31–33).

Based on these considerations, this study aimed to systematically analyze AEs associated with JAK inhibitors (deucravacitinib, upadacitinib, and tofacitinib) in patients with psoriasis and PsA as an integrated cohort using FAERS post-marketing surveillance data, while also conducting indication-stratified analyses to clarify potential differences between the two diseases.

## 2 Methods

### 2.1 Data source

During the clinical trials of drugs and after the drugs are approved for marketing, the FDA accepts voluntary ICSRs from consumers, manufacturers, and medical professionals regarding AEs, medication errors, and product quality issues. These reports are uploaded to the publicly accessible FAERS database [19]. The FAERS database of ICSRs contains seven types of data files, including demographic and administrative information (DEMO), drug information (DRUG), drug indications (INDI), patient outcomes (OUTC), adverse events (REAC), source of reports (RPSR), and the start and end dates of the drug treatment (THER). These data are interconnected in the FAERS database through specific identification numbers, such as PRIMARYID and CASEID. The FAERS database is updated quarterly and can be accessed for free on the website of the FDA (https://fis.fda.gov/extensions/fpd-qde-faers/fpd-qde-faers.html).

### 2.2 Data collection and processing

This study utilized the worldwide FAERS database from Q4–2014 to Q3–2024 to identify AEs in psoriasis and PsA treatment associated with JAK inhibitors as the primary suspects (PS) and secondary suspects (SS). All data were anonymized, and no personal information or privacy of patients would be disclosed. We used generic and brand names to identify AEs associated with JAK inhibitors, including deucravacitinib (Sotyktu), upadacitinib (Rinvoq) and tofacitinib (Xeljanz). For duplicate data, the variable matching method recommended by the FDA guidance in 2014 was adopted for elimination (34). When the CASEID was the same, the latest FDA_DT was selected. Moreover, for reports with the same CASEID and FDA_DT, the report with the largest PRIMARYID was selected.

A total of 15,488,768 reports were extracted from the FAERS database to a MySQL database using SQLiteStudio (version 3.4.9) for data screening and cleaning. We restricted the study population to psoriasis and PsA patients and ultimately obtained 4,922,570 reports. Restricting the study population to the same indication is a commonly used method in pharmacovigilance analysis. This approach enables the examination of the impact of the underlying disease and helps to reduce research bias (35).

Descriptive analyses were performed for patient demographics (sex, age), reporting countries, reporter types, report years, and clinical outcomes. Categorical variables were presented as frequencies and percentages, while continuous variables were summarized using the median with interquartile range (IQR). Meanwhile, binomial distribution tests of the clinical characteristics (sex, age and reporting country) were conducted to assess whether their distributions differed significantly between groups. Time to onset was defined as the time period from the start of drug use to the occurrence of an AE. It was calculated using the formula “EVENT_DT-START_DT + 0.5” (36). Missing variables were not included in the descriptive analysis. All AEs were coded according to the Medical Dictionary for Regulatory Activities (MedDRA) version 27.1. The characteristics of AEs were systematically analyzed at the preferred term (PT) and system organ class (SOC) levels, while Standardized MedDRA Queries (SMQs) version 28.0 were simultaneously applied to detect AEs and validate the research findings. In addition, signal differences between psoriasis and PsA were explicitly compared at the SOC level to identify potential condition-specific safety profiles.

### 2.3 Disproportionality analysis

In this study, we conducted 2 dimensionality (2D) disproportionality analyses (37, 38) using both the reporting odds ratio (ROR) and information component (IC) methods to identify safety signals for each JAK inhibitor. When reports involved multiple drugs and/or multiple AEs, we used the report unit instead of the drug-event combination.2×2 contingency tables were employed to calculate ROR and IC values with 95% confidence intervals (CIs). To reduce false negative signals, we employed statistical shrinkage transformation. The specific calculation formulas for ROR and IC are presented in **Supplementary Table S1** (39). The signal was recognized when the number of AEs was at least 3. A positive signal was identified when both the lower limit of the 95% confidence interval for ROR (ROR_025_) was greater than 1 and the lower limit of the 95% confidence interval for IC (IC_025_) exceeded 0.

### 2.4 Sensitivity analysis

To reduce the bias that demographics may cause to the research results, we separately took the data of the three drugs as a total dataset, and used multivariate logistic regression to include age and sex as independent variables to adjust the ROR and to conduct a comparison among the three drugs simultaneously for SOC signals with higher reporting rates. We also conducted interactions between age/sex/reporting country and the administration of the drug respectively to evaluate clinical factors’ additional influence on the risk of AEs. Cases with missing age/sex/reporting country information were excluded from the logistic regression analysis. In the logistic regression models, age was divided into a binary categorical variable based on the median age of the total population reporting on the three drugs, and reporting country was classified as the United States (US) versus non-US countries.

Data extraction in this study was performed using SQLiteStudio (version 3.4.9), while statistical analyses were conducted with IBM SPSS (version 25) and Python (version 3.12). All data processing steps were independently executed by two co-authors to ensure reproducibility.

## 3 Results

### 3.1 Clinical characteristics

**Figure 1:**
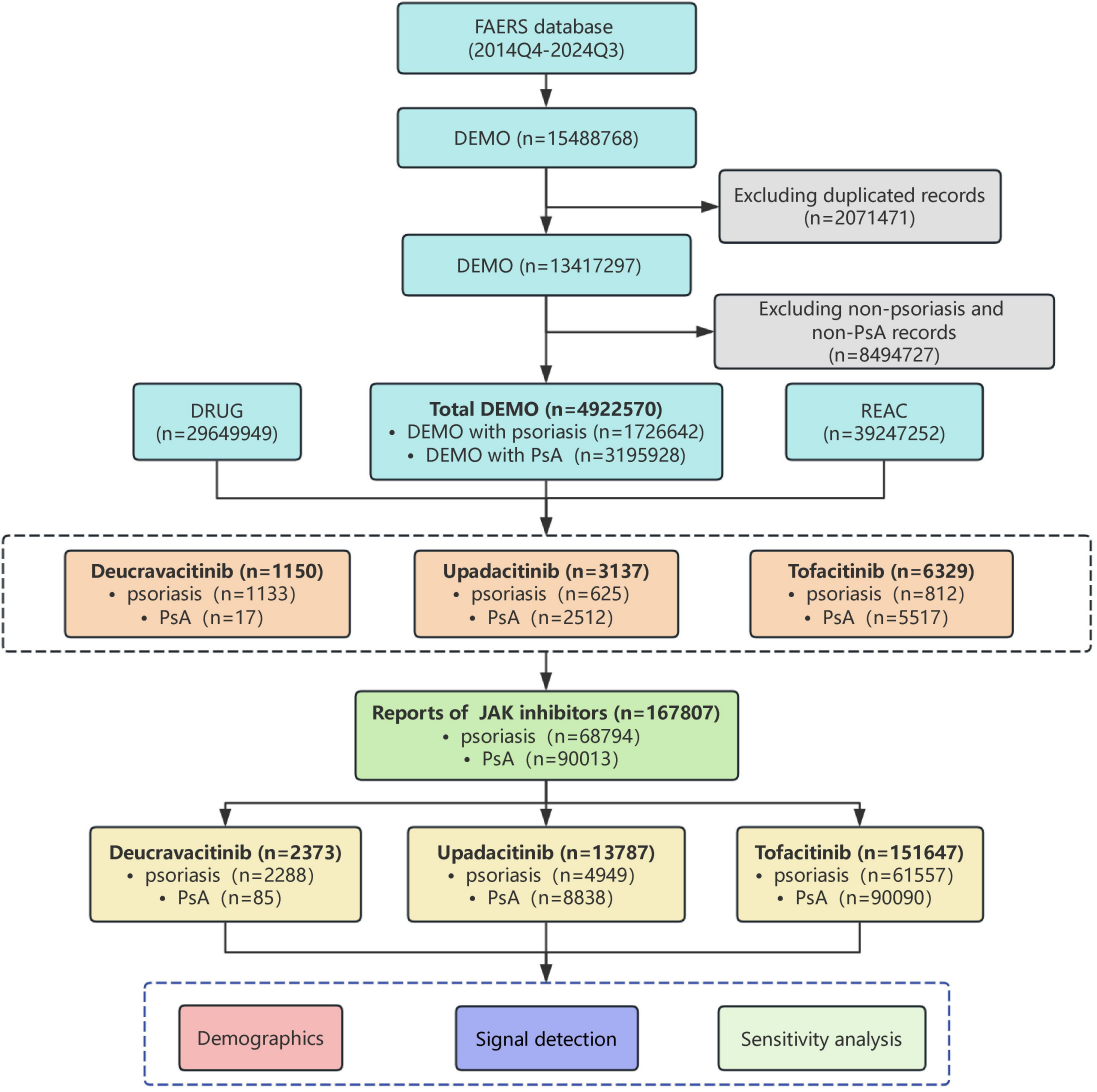
The flow diagram of selecting JAK inhibitors related adverse events in psoriasis and psoriatic arthritis patients from FAERS database.

Upon implementation of this deduplication algorithm, 2,071,471 suspected duplicate reports, representing 13.37% of the initial dataset, were excluded. Subsequent restriction to the relevant indications yielded a final cohort of 4,922,570 reports pertaining to psoriasis and PsA. A total of 167,807 reports(psoriasis: PsA=68,794:90,013) of JAK inhibitors from 10,616 cases were identified, including deucravacitinib (2,373 reports from 1,150 cases; annualized reporting rate: 206 reports per 100 person-years from 2022 to 2024), upadacitinib (13,787 reports from 3,137 cases; annualized reporting rate: 440 reports per 100 person-years from 2021 to 2024), and tofacitinib (151,647 reports from 6,329 cases; annualized reporting rate: 2,395 reports per 100 person-years from 2017 to 2024) (**Figure 1**).

The clinical characteristics of reports with JAK inhibitors from the FAERS database are shown in **Table 1**. Among the three JAK inhibitors, the majority of patients were female, with a significant difference in sex distribution between males and females (p<0.001), and the age distributions of the patients were similar, clustered mainly in 45–64 years. The median age was approximately 57 years. The US accounted for the largest proportion among the reporting countries (p<0.001). AEs for deucravacitinib and tofacitinib were predominantly reported by health professionals (44.57%, 42.18%), whereas upadacitinib reports originated primarily from consumers (82.32%). Due to the earlier market launch of tofacitinib, AEs for it were reported earlier and in greater numbers, while reports for deucravacitinib and upadacitinib were primarily concentrated in 2022-2024. The most frequent clinical outcomes of three JAK inhibitors were hospitalization followed by death.

**Table 1:** Clinical characteristics of reports with JAK inhibitors in psoriasis and psoriatic arthritis patients from the FAERS database.

Characteristic		Cases, N (%)		
		Deucravacitinib	Upadacitinib	Tofacitinib
Total cases		1150	3137	6329
Sex	Missing Data	89(7.74)	136(4.34)	191(3.02)
	Male	386(33.56)	854(27.22)	1624(25.66)
	Female	675(58.70)	2147(68.44)	4514(71.32)
	P values	<0.001	<0.001	<0.001
Age	Missing Data	274(23.83)	1648(52.53)	642(10.14)
	<18	3(0.26)	2(0.06)	18(0.28)
	18-44	243(21.13)	248(7.91)	1095(17.30)
	45-64	389(33.83)	845(26.94)	3121(49.31)
	65-74	148(12.87)	305(9.72)	1114(17.60)
	>74	93(8.09)	89(2.84)	339(5.36)
	Median (IQR)	55(40,65)	58(49,65)	57(48,65)
	P values	0.237	0.001	0.015
Reporting countries	Missing Data	0(0)	227(7.24)	6(0.09)
	1	US 1068(92.87)	US 2400(76.50)	US 4954(78.27)
	2	JP 55(4.78)	CA 152(4.85)	CA 917(14.49)
	3	AU 8(0.70)	DE 110(3.51)	GB 117(1.85)
	P values	<0.001	<0.001	<0.001
Reporters	Missing Data	17(1.48)	326(10.39)	314(4.96)
	Health professionals	505(43.91)	149(4.75)	2537(40.08)
	Consumer	334(29.04)	2314(73.76)	2224(35.14)
	Physician	256(22.26)	326(10.39)	722(11.41)
	Other health professionals	/	1(0.03)	400(6.32)
	Pharmacist	38(3.30)	21(0.67)	122(1.93)
	Lawyer	/	/	10(0.16)
Report years	Missing Data	0(0)	0(0)	0(0)
	2014(Q4)	/	/	19(0.30)
	2015	/	/	49(0.77)
	2016	/	/	79(1.25)
	2017	/	/	69(1.09)
	2018	/	/	330(5.21)
	2019	2(0.17)	9(0.29)	586(9.26)
	2020	2(0.17)	23(0.73)	1228(19.40)
	2021	/	91(2.90)	1035(16.35)
	2022	73(6.35)	1084(34.56)	1316(20.79)
	2023	679(59.04)	1140(36.34)	882(13.94)
	2024(Q1-Q3)	394(34.26)	790(25.18)	736(11.63)
Outcomes	Missing Data	973(84.61)	1458(46.48)	3652(57.70)
	Hospitalized	48(4.17)	568(18.11)	613(9.69)
	Death	13(1.13)	54(1.72)	394(6.23)
	Life-threatening	5(0.43)	20(0.64)	65(1.03)
	Disability	3(0.26)	8(0.26)	39(0.62)
	Other outcomes	108(9.39)	1029(32.80)	1566(24.74)

IQR, interquartile range; AU, Austria; CA, Canada; DE, Germany; GB, United Kingdom; JP, Japan; US,United States. P values for sex: Calculated using a binomial distribution test to assess statistical differences in sex distribution between males and females. P values for age: Calculated using a binomial distribution test to assess statistical differences in age distribution between the <57 years and≥57 years groups. P values for reporting countries: Calculated using a binomial distribution test to assess statistical differences between the US and non-US groups.

**Figure 2:**
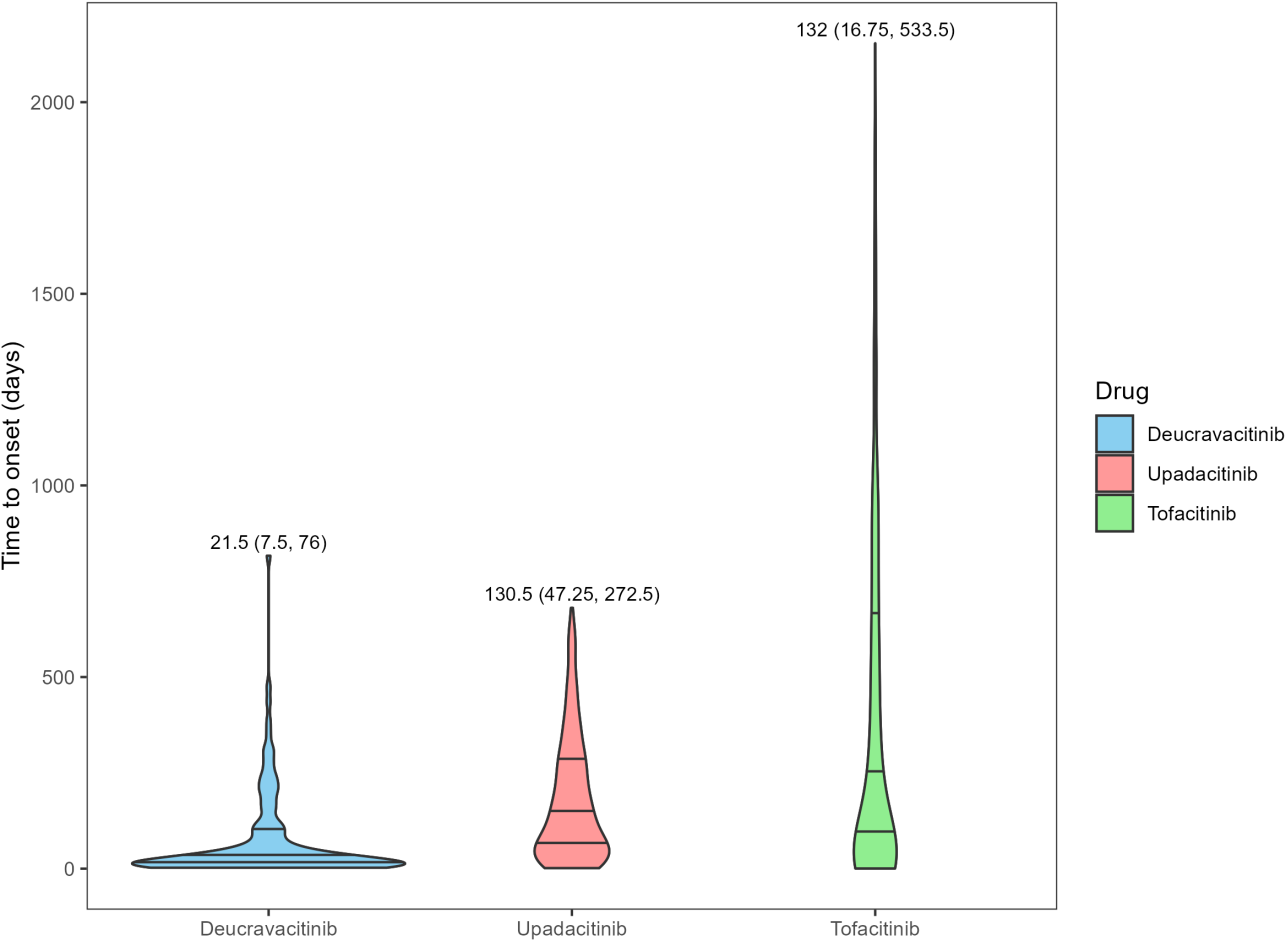
Time to onset of adverse events for different JAK inhibitors in psoriasis and psoriatic arthritis patients. The numbers in the figure represent the median and the interquartile range of time to onset.

After ranking the time to onset of AEs, the data within the middle 90% were retained, and the results are presented in **Figure 2**. Deucravacitinib demonstrated the shortest median time to onset of AEs at 21.5 days (IQR: 7.5–76 days), followed by upadacitinib (130.5 days, IQR: 47.25-272.5 days) and tofacitinib (132 days, IQR: 16.75-533.5 days). The differences in the time to onset may be partly attributed to the differences in the market launch times of the three drugs.

### 3.2 Signal detection results at the PT/SOC level

Our analysis identified 396 PT level signals across the three JAK inhibitors (**Supplementary Figure S1**, **Supplementary Tables S2-S4**). At the SOC level, the disproportionality analysis revealed distinct safety profiles as summarized in **Table 2**. The analysis results of the IC method were basically consistent with those of the ROR.

**Table 2:** Results of the signal detection at the SOC level.

SOC	Deucravacitinib			Upadacitinib			Tofacitinib		
	N	IC (95% CI)	ROR (95%CI)	N	IC (95% CI)	ROR (95%CI)	N	IC (95% CI)	ROR (95%CI)
General disorders and administration site conditions	393	0.00(-0.16, 0.12)	1.00(0.90, 1.12)	2212	-0.04(-0.11,0.01)	0.97(0.93, 1.02)	29392	0.23(0.21, 0.24) ^*^	1.17(1.16, 1.19) ^*^
Musculoskeletal and connective tissue disorders	82	-0.50(-0.87, -0.24)	0.70(0.57, 0.88)	2062	1.61(1.53, 1.66) ^*^	3.04(2.90, 3.19) ^*^	25485	1.77(1.75, 1.79) ^*^	3.42(3.38, 3.47) ^*^
Injury, poisoning and procedural complications	93	-1.20(-1.54, -0.95)	0.44(0.35, 0.54)	1015	-0.29(-0.40, -0.22)	0.82(0.77, 0.87)	17976	0.39(0.37, 0.41) ^*^	1.31(1.29, 1.33) ^*^
Gastrointestinal disorders	257	0.48(0.28, 0.63) ^*^	1.40(1.23, 1.59) ^*^	1077	0.01(-0.09, 0.09)	1.01(0.95, 1.08)	14884	0.34(0.32, 0.36) ^*^	1.27(1.25, 1.29) ^*^
Investigations	60	-0.90(-1.32, -0.59)	0.54(0.42,0.69)	786	0.27(0.15,0.36) ^*^	1.21(1.12,1.30) ^*^	11135	0.64(0.61, 0.66) ^*^	1.55(1.52, 1.58) ^*^
Infections and infestations	239	1.11(0.90, 1.26) ^*^	2.16(1.89, 2.47) ^*^	1430	1.15(1.07, 1.22) ^*^	2.23(2.11, 2.35) ^*^	9872	0.48(0.45, 0.51) ^*^	1.40(1.37, 1.43) ^*^
Skin and subcutaneous tissue disorders	753	2.63(2.51, 2.72) ^*^	6.21(5.70, 6.77) ^*^	1037	0.56(0.46, 0.64) ^*^	1.48(1.39, 1.57) ^*^	9562	0.31(0.27, 0.33) ^*^	1.24(1.21, 1.26) ^*^
Nervous system disorders	126	-0.37(-0.66, -0.15)	0.78(0.65, 0.93)	720	-0.39(-0.52, -0.30)	0.76(0.71, 0.82)	6812	-0.61(-0.65, -0.58)	0.66(0.64, 0.67)
Psychiatric disorders	40	-1.31(-1.84, -0.94)	0.40(0.29,0.55)	410	-0.50(-0.67, -0.38)	0.71(0.64,0.78)	4645	-0.46(-0.51, -0.43)	0.73(0.71, 0.75)
Respiratory, thoracic and mediastinal disorders	102	0.02(-0.31, 0.26)	1.01(0.83, 1.24)	540	-0.11(-0.26, -0.01)	0.92(0.85, 1.01)	3947	-0.70(-0.76, -0.66)	0.61(0.60, 0.63)
Metabolism and nutrition disorders	22	-0.84(-1.55, -0.34)	0.56(0.37, 0.85)	246	0.09(-0.12, 0.25)	1.07(0.94, 1.21)	3224	0.35(0.29, 0.39) ^*^	1.27(1.23, 1.32) ^*^
Immune system disorders	24	0.08(-0.61, 0.56)	1.05(0.70, 1.57)	173	0.39(0.14, 0.57) ^*^	1.31(1.13, 1.52) ^*^	3141	1.11(1.05, 1.15) ^*^	2.16(2.08, 2.24) ^*^
Surgical and medical procedures	26	-0.04(-0.70, 0.42)	0.97(0.66, 1.43)	784	2.33(2.21, 2.42) ^*^	5.03(4.68, 5.41) ^*^	1602	-0.09(-0.18, -0.03)	0.94(0.89, 0.98)
Vascular disorders	19	-1.00(-1.77, -0.47)	0.50(0.32, 0.78)	195	-0.20(-0.44, -0.03)	0.87(0.76, 1.00)	2083	-0.24(-0.32, -0.19)	0.85(0.81, 0.88)
Hepatobiliary disorders	10	-0.48(-1.55, 0.25)	0.72(0.39,1.34)	123	0.58(0.29, 0.80) ^*^	1.50(1.26,1.79) ^*^	2134	1.24(1.17, 1.30) ^*^	2.37(2.27,2.47) ^*^
Cardiac disorders	13	-1.68(-2.62, -1.04)	0.31(0.18, 0.54)	190	-0.39(-0.63, -0.21)	0.76(0.66, 0.88)	1635	-0.74(-0.82, -0.68)	0.60(0.57, 0.63)
Neoplasms benign, malignant and unspecified (incl cysts and polyps)	17	-1.46(-2.28, -0.89)	0.36(0.23, 0.59)	231	-0.26(-0.48, -0.10)	0.83(0.73, 0.95)	827	-1.88(-2.00, -1.80)	0.27(0.25, 0.29)
Eye disorders	30	-0.34(-0.95, 0.09)	0.79(0.55,1.13)	151	-0.55(-0.82, -0.36)	0.68(0.58, 0.80)	676	-1.85(-1.97, -1.76)	0.28(0.26, 0.30)
Social circumstances	5	-0.56(-2.13, 0.42)	0.68(0.28,1.63)	120	1.43(1.12,1.65) ^*^	2.69(2.25,3.22) ^*^	714	0.55(0.43,0.64) ^*^	1.46(1.36, 1.58) ^*^
Pregnancy, puerperium and perinatal conditions	/	/	/	11	-0.50(-1.52, 0.20)	0.71(0.39, 1.28)	655	1.92(1.79,2.01) ^*^	3.78(3.49, 4.08) ^*^
Renal and urinary disorders	26	-0.57(-1.23, -0.11)	0.67(0.46, 0.99)	97	-1.22(-1.55, -0.97)	0.43(0.35,0.53)	233	-3.41(-3.63, -3.26)	0.09(0.08, 0.11)
Blood and lymphatic system disorders	6	-2.19(-3.61, -1.28)	0.22(0.10, 0.49)	80	-1.08(-1.45, -0.82)	0.47(0.38, 0.59)	234	-3.00(-3.21, -2.84)	0.13(0.11, 0.14)
Ear and labyrinth disorders	16	0.73(-0.12, 1.31)	1.65(1.01, 2.70)	43	-0.35(-0.86, 0.01)	0.78(0.58, 1.06)	236	-1.36(-1.57, -1.20)	0.39(0.34, 0.44)
Product issues	5	-1.27(-2.83, -0.28)	0.42(0.17, 1.00)	11	-2.70(-3.72, -2.00)	0.15(0.09,0.28)	223	-1.87(-2.09, -1.71)	0.27(0.24, 0.31)
Endocrine disorders	1	-1.41(-5.20, 0.27)	0.38(0.05, 2.67)	8	-1.29(-2.50, -0.49)	0.41(0.20,0.82)	164	-0.44(-0.70, -0.26)	0.73(0.63, 0.86)
Congenital, familial and genetic disorders	/	/	/	5	-0.28(-1.84, 0.70)	0.82(0.34, 1.98)	99	0.54(0.20,0.78) ^*^	1.45(1.19, 1.77) ^*^
Reproductive system and breast disorders	8	-0.03(-1.24, 0.78)	0.98(0.49, 1.96)	30	-0.65(-1.26, -0.22)	0.64(0.44, 0.91)	57	-3.18(-3.62, -2.87)	0.11(0.08, 0.14)

N, The number of reports for SOC associated with study drugs. The asterisk (*) indicates statistical significance.

For deucravacitinib, we identified 3 significant SOC signals and 61 significant PT signals. The significant SOC signals were skin and subcutaneous tissue disorders (IC_025_ = 2.51), infections and infestations (IC_025_ = 0.90), and gastrointestinal disorders (IC_025_ = 0.28). The predominant PT signals included acne (IC_025_ = 4.90), folliculitis (IC_025_ = 4.80), mouth ulceration (IC_025_ = 4.19), and aphthous ulcer (IC_025_ = 3.96).

Analysis of upadacitinib revealed 8 significant SOC signals and 229 significant PT signals. The primary SOC signals comprised musculoskeletal and connective tissue disorders (IC_025_ = 1.53), infections and infestations (IC_025_ = 1.07), and skin and subcutaneous tissue disorders (IC_025_ = 0.46). Key PT signals were subcutaneous drug absorption impaired (IC_025_ = 5.53), adjustment disorder with depressed mood (IC_025_ = 4.76), swollen joint count increased (IC_025_ = 4.65), pustular psoriasis (IC_025_ = 4.57).

Tofacitinib exhibited the highest number of signals, with 13 significant SOC signals and 262 significant PT signals. The predominant SOC signals were pregnancy, puerperium and perinatal conditions (IC_025_ = 1.79), musculoskeletal and connective tissue disorders (IC_025_ = 1.75), and hepatobiliary disorders (IC_025_ = 1.17). The PTs with the highest disproportionality estimates included swollen joint count increased (IC_025_ = 6.82), rheumatic fever (IC_025_ = 6.75), facet joint syndrome (IC_025_ = 6.36), and deep vein thrombosis postoperative (IC_025_ = 6.34).

Overall, skin and subcutaneous tissue disorders, infections and infestations, and gastrointestinal disorders were frequently reported AEs for JAK inhibitors. Musculoskeletal and connective tissue disorders showed higher reporting rates for upadacitinib and tofacitinib. These signals are expected adverse reactions as stated in the prescribing information. Comparative analysis with prescribing information revealed previously undocumented AEs associated with deucravacitinib, including myalgia (IC_025_ = 0.79) and blood creatine phosphokinase increased (IC_025_ = 0.02). Additionally, we also found unexpected nervous system disorder signals for upadacitinib such as migraine (IC_025_ = 1.17) and amnesia (IC_025_ = 1.13), and psychiatric disorder signals including adjustment disorder with depressed mood (IC_025_ = 4.76) and sleep disorder due to general medical condition, insomnia type (IC_025_ = 4.03). In the subgroup signal analysis stratified by indication (**Supplementary Figure S2**), most AE signals were generally comparable between psoriasis and PsA. However, upadacitinib demonstrated a higher positive signal for metabolism and nutrition disorders in psoriasis patients compared with PsA patients.

### 3.3 Signal detection results at the SMQ level

**Figure 3:**
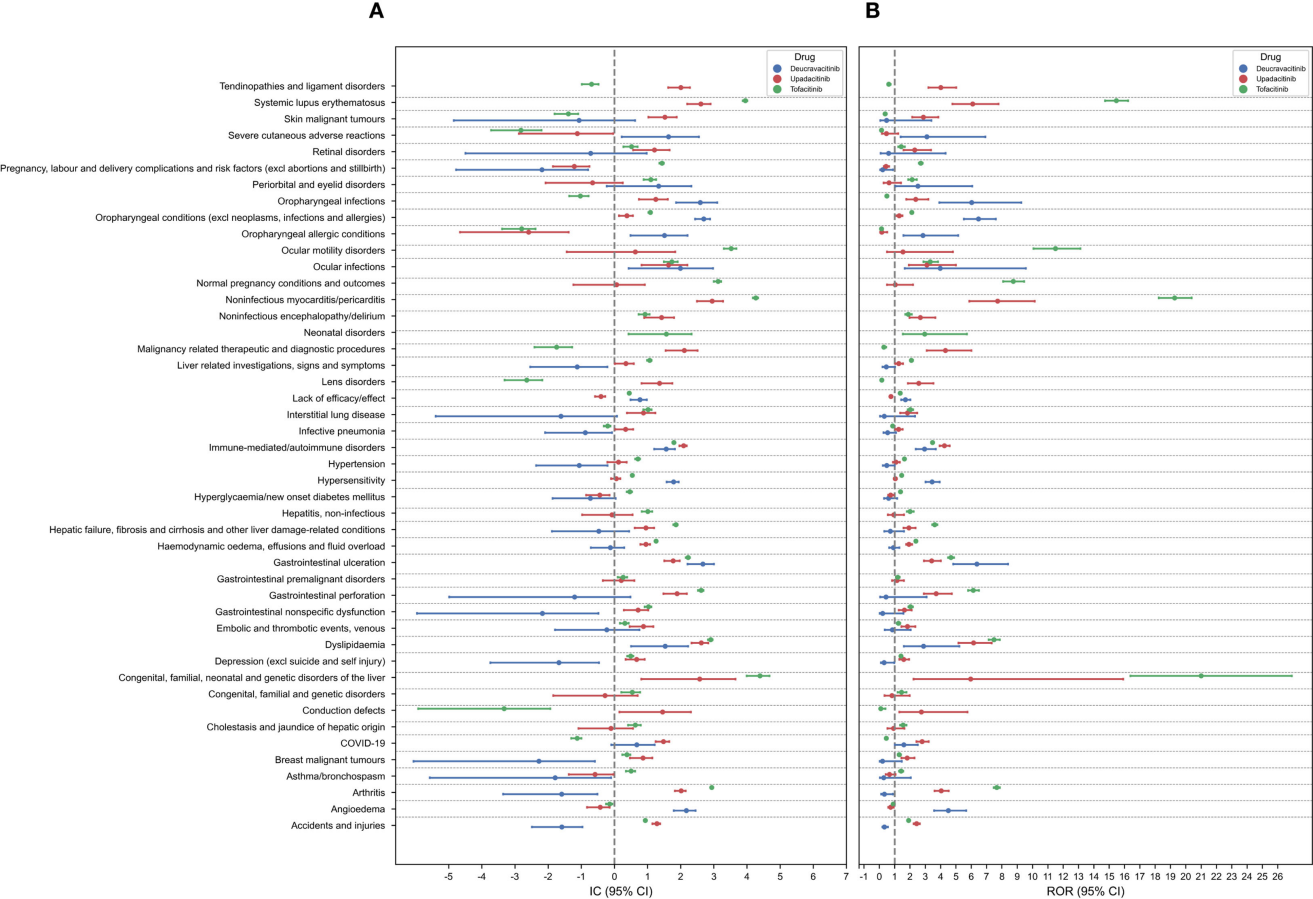
Forest plots of the signal detection results at the SMQ level. **(A)** IC (95% CI). **(B)** ROR (95% CI).

The AEs at the PT level were clustered to the SMQ level, and the results are shown in **Figure 3**. A total of 46 positive SMQ signals were found for the three drugs. The predominant positive SMQ signals for deucravacitinib included oropharyngeal conditions (IC_025_ = 2.44), gastrointestinal ulceration (IC_025_ = 2.21), oropharyngeal infections (IC_025_ = 1.86), and angioedema (IC_025_ = 1.79). Upadacitinib demonstrated significant SMQ signals for noninfectious myocarditis/pericarditis (IC_025_ = 2.49), dyslipidemia (IC_025_ = 2.33), systemic lupus erythematosus (IC_025_ = 2.20), and immune-mediated/autoimmune disorders (IC_025_ = 1.96). Notably, tofacitinib was associated with the highest disproportionality scores at the SMQ level, particularly for noninfectious myocarditis/pericarditis (IC_025_ = 4.18), congenital/familial/neonatal genetic liver disorders (IC_025_ = 3.99), systemic lupus erythematosus (IC_025_ = 3.87), and ocular motility disorders (IC_025_ = 3.30).

Upadacitinib and tofacitinib were associated with higher reporting rates for systemic lupus erythematosus, arthritis and noninfectious myocarditis/pericarditis compared to deucravacitinib. Consistent with the analysis at the SOC level, deucravacitinib showed dermatologic AEs signals exceeding those of upadacitinib and tofacitinib. Positive signals of gastrointestinal ulceration, dyslipidemia, immune-mediated/autoimmune disorders, oropharyngeal conditions, and ocular infections were present in all three drugs. The AE signals at the SOC level were generally consistent with those at the SMQ level, validating the results of the AEs data mining in this study and confirming the accuracy and reliability of our findings.

### 3.4 Sensitivity analysis

After excluding data with missing age/sex/reporting country information, the final data used for sensitivity analysis included deucravacitinib (860 cases), upadacitinib (1,475 cases), and tofacitinib (5,680 cases). Three common SOC signals (skin and subcutaneous tissue disorders, infections and infestations, and gastrointestinal disorders) for JAK inhibitors were selected to conduct logistic regression analysis of clinical factors. Age/sex/reporting country were included as binary variables in the logistic regression model. As a relative indicator, ROR is influenced by the background reporting rate (14).Since our main objective is to conduct a comparison among the three drugs, when we used the data of the three JAK inhibitors for psoriasis and PsA as an additional dataset for sensitivity analysis, there would be certain differences between the ROR results and the previous disproportionality analysis results.

The logistic regression analysis, after adjusting for age and sex, revealed distinct AE profiles among the JAK inhibitors (Model 2 in **Table 3**). Deucravacitinib demonstrated significantly higher positive signal for skin and subcutaneous tissue disorders (adjusted ROR:4.33, 95%CI:3.73-5.04) compared to both upadacitinib and tofacitinib. Upadacitinib and tofacitinib were associated with greater susceptibility to infectious diseases (upadacitinib [adjusted ROR:1.15, 95%CI:1.02-1.29], tofacitinib [adjusted ROR:1.23, 95%CI:1.11-1.37]). Tofacitinib additionally showed noteworthy gastrointestinal disorders signals (adjusted ROR:1.67, 95%CI:1.46-1.91).

**Table 3:** Results of the sensitivity analysis of different JAK inhibitors.

Drug	SOC	Crude ROR (95%CI)	Adjusted ROR (95%CI)	ROR (95%CI) of subgroup comparison		
		Model 1	Model 2	Model 3	Model 4	Model 5
Deucravacitinib	Skin and subcutaneous tissue disorders	4.24 (3.65-4.91)	4.33 (3.73-5.04)	1.09 (0.79-1.50)	0.80 (0.59-1.08)	5.91 (3.21-10.89)
	Infections and infestations	0.45 (0.38-0.54)	0.47 (0.39-0.56)	0.79 (0.54-1.16)	1.14 (0.79-1.63)	0.57 (0.32-1.03)
	Gastrointestinal disorders	0.82 (0.68-1.00)	0.84 (0.69-1.02)	0.85 (0.56-1.29)	1.63 (1.11-2.40)	7.10 (2.50-20.13)
Upadacitinib	Skin and subcutaneous tissue disorders	0.46 (0.39-0.54)	0.46 (0.39-0.55)	0.76 (0.52-1.12)	1.06 (0.75-1.51)	2.27 (1.56-3.31)
	Infections and infestations	1.14 (1.02-1.29)	1.15 (1.02-1.29)	1.02 (0.77-1.35)	1.15 (0.91-1.47)	3.50 (2.66-4.61)
	Gastrointestinal disorders	0.53 (0.45-0.63)	0.53 (0.45-0.63)	0.74 (0.50-1.10)	1.38 (0.98-1.94)	3.55 (2.44-5.16)
Tofacitinib	Skin and subcutaneous tissue disorders	0.69 (0.62-0.78)	0.69 (0.61-0.77)	1.41 (1.08-1.83)	1.18 (0.93-1.49)	0.12 (0.09-0.16)
	Infections and infestations	1.25 (1.12-1.39)	1.23 (1.11-1.37)	0.98 (0.77-1.25)	0.81 (0.65-1.00)	0.48 (0.37-0.62)
	Gastrointestinal disorders	1.69 (1.47-1.94)	1.67 (1.46-1.91)	1.35 (0.99-1.84)	0.65 (0.50-0.86)	0.21 (0.15-0.29)

Model 1, crude ROR.

Model 2, adjusted ROR for sex and age.

Model 3, ROR of the interaction term between sex and the drug taking males as the reference via a logistic regression.

Model 4, ROR of the interaction term between age and the drug taking patients<57 years as the reference via a logistic regression.

Model 5, ROR of the interaction term between reporting countries and the drug taking non-US countries as the reference via a logistic regression.

For female patients taking tofacitinib, the positive signals of skin and subcutaneous tissue disorders were higher than those in male patients (Model 3 in **Table 3**). Patients aged ≥57 years exhibited significantly higher gastrointestinal disorder signals with deucravacitinib. Conversely, for patients aged <57 years using tofacitinib, the signals of gastrointestinal disorders were more evident (Model 4 in **Table 3**). No obvious differences in terms of age and sex were observed for the remaining AEs. The sensitivity analysis stratified by reporting country revealed substantial differences in the positive AE signals between reports originating from the US and those from non-US countries (Model 5 in **Table 3**).

## 4 Discussion

The development of targeted therapies for psoriasis and PsA has significantly expanded treatment options, yet accompanying safety concerns require rigorous evaluation. This study systematically assesses the AE profiles of JAK inhibitors using FAERS data, providing critical post-marketing safety evidence to optimize risk-benefit decisions in clinical practice.

There have been some analyses of AEs regarding JAK inhibitors in previous studies (40, 41). However, limiting the indications is a commonly used method in pharmacovigilance analysis. Currently, no comprehensive safety assessment has been conducted on JAK inhibitors in psoriasis and PsA, and no direct comparative analysis between the two indications has been performed. To our knowledge, this is the most extensive and comprehensive pharmacovigilance study of JAK inhibitors used for psoriasis and PsA, providing certain references in clinical practice.

As a first-generation JAK inhibitor, tofacitinib exhibits broad activity across multiple JAK isoforms. Our analysis revealed a higher positive frequency of safety signals with tofacitinib compared to upadacitinib and deucravacitinib, consistent with its lower selectivity and longer post-marketing surveillance period. In contrast, upadacitinib (JAK1-selective) and deucravacitinib (TYK2-specific) demonstrated targeted kinase inhibition profiles that correlated with improved safety outcomes (42). And deucravacitinib exhibited fewer significant AE signals compared with upadacitinib and tofacitinib in this study. These distinct pharmacological properties may explain their differential AE profiles. Notably, deucravacitinib represents the first oral TYK2 inhibitor approved for psoriasis treatment, marking a significant advancement in immunomodulatory therapy. Its unique allosteric inhibition mechanism not only enhances treatment specificity but also shows promising translational potential for other immune-mediated diseases including PsA and systemic lupus erythematosus (21, 43).

Our analysis identified infections and infestations as class-wide AEs across all three JAK inhibitors. While these drugs effectively mitigate inflammation through pathway inhibition, their immunosuppressive properties may concurrently impair host defense mechanisms, potentially triggering paradoxical inflammatory reactions (44). Patients should be guided to seek medical help when encountering severe infections, and the discontinuation of JAK inhibitors should be considered. Due to its highly selective mechanism of action, deucravacitinib was associated with fewer reporting signals for infections and infestations compared to other JAK inhibitors. Furthermore, the positive signals for immune system disorders, hepatobiliary disorders, and musculoskeletal and connective tissue disorders were significantly lower with deucravacitinib than with tofacitinib and upadacitinib. However, patients treated with deucravacitinib exhibited a notably higher signal of skin and subcutaneous tissue disorders (e.g., acne, skin burning sensation, erythema, pruritus). The underlying mechanism remains unclear but may be related to immune-mediated alterations in the skin microbiome induced by deucravacitinib therapy (45). Since JAK inhibitors are administered orally, gastrointestinal-related AEs will accompany their use. Sensitivity analysis revealed that tofacitinib had higher gastrointestinal disorders signals than the other two drugs. In clinical practice, gastrointestinal disorder is the most common non-serious AE leading to tofacitinib discontinuation (46, 47).

SOC level analysis revealed pharmacovigilance signals for musculoskeletal and connective tissue disorders with upadacitinib and tofacitinib which were similar to the findings of other studies (48). The underlying mechanism may be related to the inhibition of JAK signaling pathways by these drugs. When JAK inhibitors interfere with these pathways, they can disrupt the normal balance of cytokines, potentially leading to abnormal tissue remodeling and inflammation in the musculoskeletal and connective tissue. Deucravacitinib showed no significant signal at this SOC level, but PT level analysis detected potential musculoskeletal AEs, including myalgia and blood creatine phosphokinase increased which were not previously documented in the prescribing information. This discrepancy is likely due to the relatively small number of musculoskeletal-related reports and the heterogeneity of PTs within this SOC, which diluted the disproportionate reporting when aggregated. Notably, this observation is also consistent with the unique pharmacological profile of deucravacitinib as a highly selective, allosteric TYK2 inhibitor (21, 43). Long-term clinical trial data for deucravacitinib have not revealed emergent and clustered musculoskeletal safety signals (45).Psoriasis and PsA patients frequently experience mood disorders such as anxiety and depression, often accompanied by pain, pruritus, and sleep disturbances (49). Moreover, some studies have found that oral anti-psoriasis drugs are significantly associated with a higher ROR of depression (50). Our study also identified new PT signals for nervous system disorders and psychiatric disorders associated with upadacitinib. These previously unreported AEs in the prescribing information warrant heightened clinical vigilance during subsequent therapeutic applications. Additionally, further research should be conducted to quantify and identify these unexpected risk factors. Notably, some AEs may not be directly attributable to the pharmacological intervention itself, but rather to the underlying disease or its complications (51). Nevertheless, these findings remain clinically noteworthy and warrant appropriate monitoring.

In the subgroup signal analysis comparing psoriasis and PsA, the overall patterns of safety signals appeared broadly similar across the two indications. However, metabolism and nutrition disorder reporting rates associated with upadacitinib were higher in psoriasis patients. Previous studies by Bostoen et al. (52) have similarly reported a higher prevalence of metabolic syndrome in psoriasis compared with PsA, a difference largely attributed to the significantly higher rates of abdominal obesity observed in psoriasis (52). Metabolic syndrome is recognized as one of the most common and clinically relevant comorbidities in psoriasis (53), and its development has been linked to several potential mechanisms, including endoplasmic reticulum stress, excessive release of proinflammatory cytokines, overproduction of reactive oxygen species, etc. (54).

Common AEs associated with JAK inhibitors include infections, neoplasms, musculoskeletal and connective tissue disorders, cardiovascular events, etc. (14, 55, 56) Zhao et al. (40) reported that the predominant AEs were related to skin and subcutaneous tissue disorders as well as infections and infestations in a study on the real-world safety of deucravacitinib. Upadacitinib was found to be associated with increased risks of respiratory events, cancer, musculoskeletal disorders, and infections (48). Tofacitinib has been linked to a higher risk of musculoskeletal disorders (48), systemic infections, tumor progression, and thromboembolic events (57). Consistent with these previous findings, our analysis revealed positive signals for musculoskeletal and connective tissue disorders, infections and skin and subcutaneous tissue disorders, while neoplasms and cardiovascular events did not reach significance at the SOC level but were observed at the PT level, including signals for breast cancer, skin cancer, peripheral venous disease, and hypertension. Several factors may explain the differences observed between our findings and those reported in previous studies. Firstly, our analysis relied on FAERS database, which predominantly captures reports from US patients, whereas some studies drew upon randomized clinical trials or global pharmacovigilance databases such as VigiBase. Differences in genetic background, comorbidities, and concomitant medications across populations are well-recognized determinants of AE distributions (58). Secondly, our study was restricted to psoriasis and PsA, whereas other studies may have examined different indications, such as rheumatoid arthritis, or may not have been limited to a specific indication (14, 48). Given the variation in underlying disease mechanisms, immune system alterations, and background therapies, the AE patterns may understandably differ across indications. Finally, even within the JAK inhibitor class, differences in kinase selectivity and binding affinity may result in heterogeneous safety profiles (59).

Among the three investigated drugs, the number of reports from female patients was significantly greater than that from male patients, potentially attributable to higher willingness to participate in pharmacovigilance systems (60) or a higher incidence rate of female patients. In this study, female patients taking tofacitinib exhibited a higher likelihood of developing skin and subcutaneous tissue disorders, which may be linked to sex-related differences in drug metabolism, immune responses, and social behaviors (41). Such sex differences highlighted the importance of considering sex-specific factors when managing and monitoring AEs in patients with psoriasis and PsA. The opposite conclusions in this study regarding the likelihood of gastrointestinal disorders in different age groups for deucravacitinib and tofacitinib may be attributed to differences in indication proportions and drug mechanisms. However, our sensitivity analysis suggested that sex and age had minimal influence on the positive signals for the remaining AEs. Real world studies remain necessary to further elucidate potential age and sex differences. In the sensitivity analysis stratified by reporting country, we observed notable differences in positive AE signals between US and non-US countries reports. Given that FAERS is a US-centric database with the majority of reports originating from the US, these findings suggest that our results may be more representative of the US population.

The analysis of the occurrence time of AEs indicated that most AEs of deucravacitinib occurred within one month and it was essential to conduct monitoring in the early stage of treatment. In contrast, the median occurrence time of AEs for upadacitinib and tofacitinib was around 130 days, which suggested that long-term attention should be paid to their adverse symptoms after medication. In interpreting the results of this study, it is important to consider potential biases arising from differences in both market duration and patient exposure among the three JAK inhibitors. To address this, we calculated annualized reporting rates (reports per 100 person-years) and applied statistical shrinkage in our disproportionality analyses (ROR and IC), which help mitigate these biases and reduce this limitation. Notably, our study revealed a substantial proportion of missing age data. As presented in **Table 1**, the extent of missing age data varied considerably across the three JAK inhibitors, with upadacitinib showing the highest rate of missingness (52.53%), followed by deucravacitinib (23.83%) and tofacitinib (10.14%). We acknowledge that the missing age data constitutes a potential source of selection bias. Prospective studies with more comprehensive clinical data will be essential to validate these results in the future. Other factors that should be considered included the relatively high proportion of non-medical professionals in the reports of upadacitinib, which was consistent with the results of other studies (14, 41) Although reports from healthcare providers may be more systematic and detailed, spontaneous reports from consumers often capture a wider range of patient experiences (31). Nevertheless, we must still give consideration to the AEs reported by consumers.

Some limitations of this research should be acknowledged. Firstly, the disproportionality analysis applied in pharmacovigilance study has issues such as lack of detailed patient exposure information, reporting biases, and confounding biases. It cannot provide the true incidence rate and make causal inferences. Hypotheses generated from this analysis require validation in subsequent real-world studies (61). To minimize bias, this study employed statistical shrinkage transformation as a corrective methodology. Secondly, the FAERS database has defects including data quality issues, incomplete clinical data, underreporting data, redundancy and missing information, lack of exposure denominators, and diverse information sources. We strive to ensure data integrity and reliability by removing duplicate data and missing values, as well as performing data extraction and preprocessing in this study. Nevertheless, important clinical variables such as comorbidities, concomitant medications, laboratory parameters and other clinical details are often incomplete and not systematically or consistently captured, which limits our ability to fully adjust for these potential confounders. Thirdly, this study relied solely on the FAERS database, which is a spontaneous reporting system primarily comprising data from the US. Consequently, the demographic homogeneity restricts the ability to fully assess the influence of racial, genetic, and regional factors on the safety profiles of JAK inhibitors. Future studies that incorporate multinational or multiethnic databases and leverage real-world data with richer clinical details are warranted. Such studies would help validate and extend these findings across diverse populations and comprehensively evaluate the influence of comorbidities and other clinical factors. Despite these limitations, this study still makes use of a large real-world database, providing valuable insights for the detection of AEs.

## 5 Conclusions

This systematic pharmacovigilance study reveals distinct AE profiles associated with JAK inhibitor use in psoriasis and PsA patients. Skin and subcutaneous tissue disorders, infections and infestations, and gastrointestinal disorders were frequently reported AE signals for JAK inhibitors. Musculoskeletal and connective tissue disorders were prominent AEs associated with upadacitinib and tofacitinib. The reporting rates of skin and subcutaneous tissue disorder AEs for deucravacitinib were higher than those for upadacitinib and tofacitinib, whereas most other AE reporting rates for deucravacitinib were lower. The unexpected PT signals related to musculoskeletal and connective tissue disorders of deucravacitinib, as well as the PT signals related to nervous system disorders and psychiatric disorders of upadacitinib, required heightened attention. Comparisons between psoriasis and PsA showed broadly consistent AE signal patterns. Subgroup analysis suggested that female subjects had a higher likelihood of developing skin and subcutaneous tissue disorders after taking tofacitinib. Since disproportionality analysis cannot infer causal relationships, the results require further validation in studies with denominator data. Clinicians should maintain heightened vigilance for potential AEs and implement enhanced patient monitoring protocols to optimize medication safety.

## Data Availability

The original contributions presented in the study are included in the article/**Supplementary Material**. Further inquiries can be directed to the corresponding author.
