# Supplementary material for: Comprehensive disproportionality analysis of individual case safety reports associated with Janus kinase inhibitors in psoriasis and psoriatic arthritis using the FAERS database
Source: Front Immunol. 2025 Oct 2;16:1629886. doi: 10.3389/fimmu.2025.1629886 (PMC12529101; doi:10.3389/fimmu.2025.1629886)
Supplement: Supplementary file 1 [file Table1.docx]

**Supplementary materials**

Table S1 The specific calculation formulas and criteria for ROR and IC

| **Method** | **Formula** | **Criteria** |
| --- | --- | --- |
| ROR | ROR=(a+0.5)/((a+b) (a+c)/(a+b+c+d) +0.5)  95%CI=$e^{\ln\left( \mathrm{ROR} \right)\pm1.96{(\frac{1}{a}+\frac{1}{b}+\frac{1}{c}+\frac{1}{d})}^{0.5}}$ | a≥3 and 95% CI (lower limit)＞1 |
| IC | IC=log_2_((a+0.5)/((a+b) (a+c)/(a+b+c+d) +0.5))  95%CI=IC-3.3${(a+0.5)}^{-0.5}-2{(a+0.5)}^{-1.5}$ | a≥3 and 95% CI (lower limit)＞0 |

a: The number of reports for target adverse event associated with study drug.

b: The number of reports for all other adverse events associated with study drug.

c: The number of reports for target adverse event associated with all other drugs.

d: The number of reports for all other adverse events associated with all other drugs.


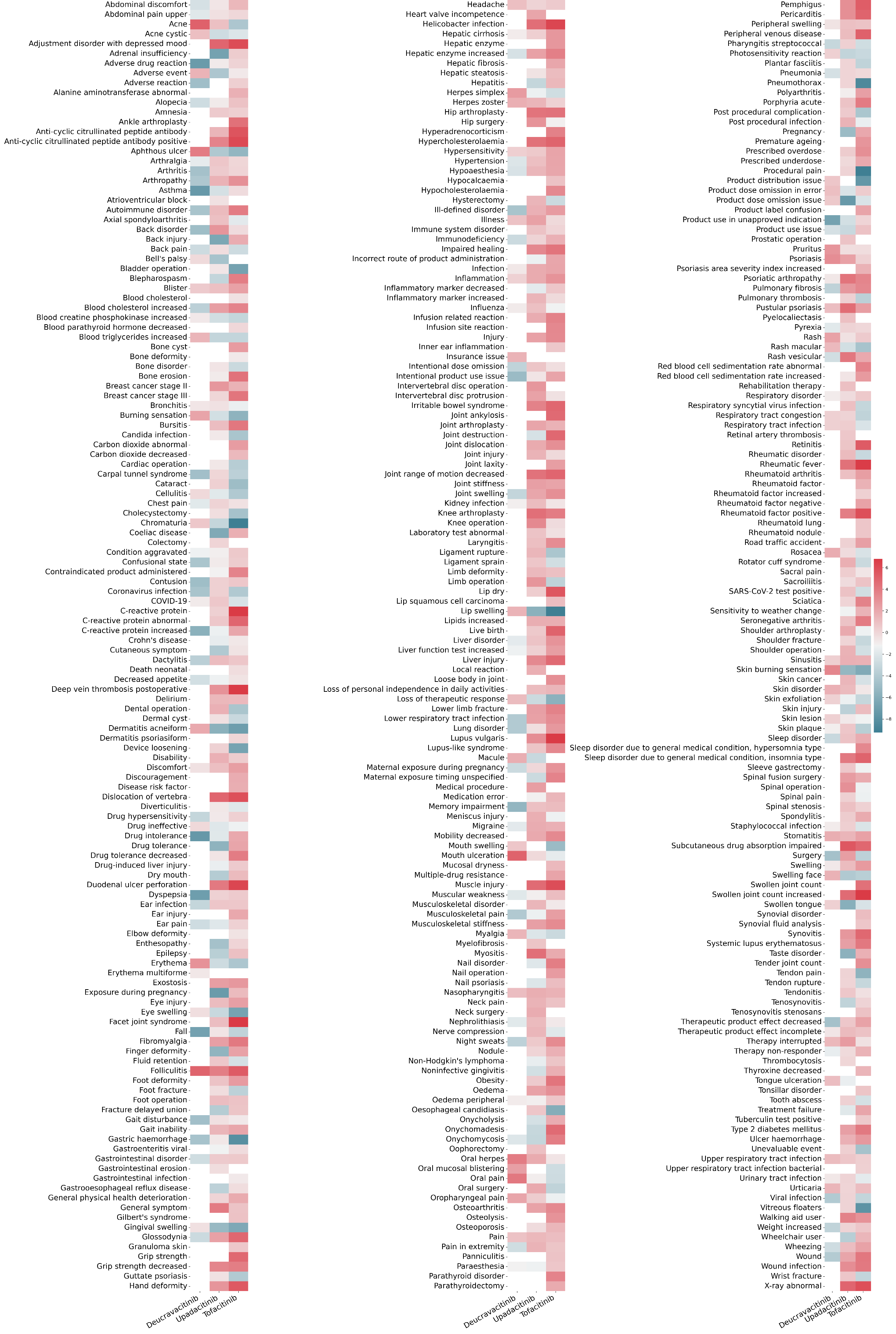


Figure S1 The heatmap of the IC_025_ values at the PT level for different JAK inhibitors

Table S2 All positive adverse events of Deucravacitinib at the PT level

| **PT** | **Report numbers** | **IC (95%CI)** | **ROR (95%CI)** |
| --- | --- | --- | --- |
| Acne | 136 | 5.19(4.90,5.39) | 36.42(30.62,43.31) |
| Folliculitis | 36 | 5.36(4.80,5.75) | 40.96(29.44,56.97) |
| Mouth ulceration | 34 | 4.76(4.19,5.17) | 27.17(19.36,38.13) |
| Aphthous ulcer | 22 | 4.67(3.96,5.17) | 25.51(16.75,38.84) |
| Skin burning sensation | 50 | 3.94(3.47,4.28) | 15.36(11.61,20.33) |
| Oral pain | 22 | 4.04(3.32,4.54) | 16.42(10.79,24.99) |
| Psoriasis | 71 | 3.58(3.18,3.86) | 11.94(9.43,15.12) |
| Oral herpes | 19 | 3.92(3.15,4.46) | 15.17(9.66,23.84) |
| Erythema | 92 | 3.49(3.14,3.74) | 11.24(9.12,13.84) |
| Pruritus | 127 | 3.15(2.85,3.36) | 8.86(7.41,10.60) |
| Burning sensation | 27 | 3.17(2.53,3.62) | 8.99(6.15,13.15) |
| Dermatitis acneiform | 8 | 3.55(2.34,4.35) | 11.72(5.85,23.49) |
| Rash | 101 | 2.57(2.24,2.81) | 5.95(4.88,7.27) |
| Adverse event | 25 | 2.64(1.97,3.11) | 6.23(4.20,9.25) |
| Blood triglycerides increased | 8 | 3.12(1.91,3.92) | 8.68(4.33,17.38) |
| Rosacea | 6 | 3.26(1.85,4.17) | 9.60(4.31,21.41) |
| Herpes simplex | 6 | 3.25(1.84,4.16) | 9.52(4.27,21.23) |
| Stomatitis | 17 | 2.57(1.76,3.14) | 5.95(3.69,9.58) |
| Oral mucosal blistering | 6 | 3.08(1.67,4.00) | 8.48(3.80,18.90) |
| Skin disorder | 11 | 2.65(1.62,3.34) | 6.26(3.46,11.32) |
| Swelling face | 15 | 2.48(1.61,3.08) | 5.59(3.36,9.29) |
| Acne cystic | 5 | 3.13(1.56,4.11) | 8.73(3.62,21.00) |
| Urticaria | 27 | 2.09(1.44,2.54) | 4.25(2.90,6.20) |
| Oropharyngeal pain | 19 | 2.20(1.43,2.74) | 4.61(2.93,7.23) |
| Therapy interrupted | 15 | 2.19(1.31,2.79) | 4.55(2.74,7.56) |
| Rash macular | 9 | 2.42(1.28,3.18) | 5.36(2.78,10.31) |
| Chromaturia | 7 | 2.53(1.22,3.38) | 5.76(2.74,12.10) |
| Blister | 11 | 2.15(1.12,2.84) | 4.42(2.45,8.00) |
| Upper respiratory tract infection | 10 | 2.20(1.12,2.92) | 4.59(2.46,8.54) |
| Pustular psoriasis | 4 | 2.88(1.11,3.96) | 7.35(2.75,19.62) |
| Tongue ulceration | 4 | 2.87(1.10,3.95) | 7.29(2.73,19.47) |
| Macule | 4 | 2.84(1.08,3.92) | 7.17(2.69,19.13) |
| Product dose omission in error | 7 | 2.35(1.05,3.20) | 5.10(2.43,10.72) |
| Herpes zoster | 11 | 2.04(1.02,2.73) | 4.11(2.27,7.44) |
| Lip swelling | 7 | 2.14(0.83,2.99) | 4.40(2.09,9.23) |
| Insurance issue | 5 | 2.38(0.82,3.36) | 5.20(2.16,12.51) |
| Myalgia | 19 | 1.56(0.79,2.10) | 2.95(1.88,4.64) |
| Product distribution issue | 4 | 2.49(0.73,3.57) | 5.62(2.11,14.99) |
| Cellulitis | 8 | 1.84(0.62,2.64) | 3.57(1.78,7.14) |
| Drug ineffective | 106 | 0.94(0.62,1.17) | 1.91(1.58,2.33) |
| Product dose omission issue | 28 | 1.20(0.57,1.65) | 2.30(1.58,3.34) |
| **PT** | **Report numbers** | **IC (95%CI)** | **ROR (95%CI)** |
| Bell's palsy | 3 | 2.63(0.56,3.83) | 6.18(1.99,19.20) |
| Loss of therapeutic response | 3 | 2.52(0.45,3.73) | 5.74(1.85,17.83) |
| Nasopharyngitis | 18 | 1.21(0.41,1.76) | 2.31(1.45,3.67) |
| Eye swelling | 6 | 1.80(0.39,2.71) | 3.49(1.56,7.77) |
| Gingival swelling | 3 | 2.44(0.37,3.64) | 5.42(1.74,16.82) |
| Headache | 44 | 0.85(0.35,1.21) | 1.80(1.34,2.43) |
| Sinusitis | 11 | 1.34(0.32,2.04) | 2.54(1.40,4.59) |
| Discomfort | 8 | 1.51(0.30,2.31) | 2.84(1.42,5.69) |
| Respiratory tract infection | 5 | 1.85(0.29,2.84) | 3.61(1.50,8.68) |
| Skin lesion | 5 | 1.85(0.29,2.83) | 3.60(1.50,8.66) |
| Swollen tongue | 5 | 1.85(0.28,2.83) | 3.59(1.49,8.64) |
| Respiratory tract congestion | 4 | 2.04(0.27,3.12) | 4.11(1.54,10.96) |
| Illness | 11 | 1.27(0.25,1.97) | 2.42(1.34,4.37) |
| Photosensitivity reaction | 4 | 2.01(0.25,3.09) | 4.03(1.51,10.75) |
| Pain | 42 | 0.73(0.21,1.09) | 1.65(1.22,2.24) |
| Skin exfoliation | 9 | 1.33(0.19,2.09) | 2.51(1.30,4.82) |
| Bronchitis | 8 | 1.35(0.14,2.15) | 2.55(1.27,5.11) |
| Mouth swelling | 3 | 2.16(0.09,3.36) | 4.46(1.44,13.85) |
| Erythema multiforme | 3 | 2.14(0.07,3.35) | 4.41(1.42,13.70) |
| Blood creatine phosphokinase increased | 4 | 1.79(0.02,2.86) | 3.45(1.29,9.20) |

Table S3 All positive adverse events of upadacitinib at the PT level

| **PT** | **Report numbers** | **IC (95% CI)** | **ROR (95%CI)** |
| --- | --- | --- | --- |
| Subcutaneous drug absorption impaired | 39 | 6.06(5.53,6.44) | 66.72(47.46,93.79) |
| X-ray abnormal | 36 | 5.48(4.92,5.87) | 44.63(31.96,62.31) |
| Dislocation of vertebra | 36 | 5.34(4.79,5.74) | 40.52(29.06,56.49) |
| Adjustment disorder with depressed mood | 36 | 5.32(4.76,5.71) | 39.93(28.64,55.66) |
| Swollen joint count increased | 42 | 5.17(4.65,5.53) | 35.90(26.43,48.76) |
| Pustular psoriasis | 39 | 5.10(4.57,5.48) | 34.28(24.95,47.09) |
| Rheumatic fever | 41 | 4.91(4.39,5.28) | 30.11(22.11,41.02) |
| Psoriatic arthropathy | 233 | 4.52(4.30,4.68) | 22.95(20.15,26.14) |
| Rash vesicular | 38 | 4.67(4.13,5.06) | 25.54(18.54,35.19) |
| Duodenal ulcer perforation | 47 | 4.53(4.05,4.88) | 23.12(17.33,30.83) |
| Sleep disorder due to general medical condition, insomnia type | 45 | 4.53(4.03,4.88) | 23.08(17.20,30.97) |
| General symptom | 40 | 4.49(3.96,4.87) | 22.48(16.46,30.71) |
| Folliculitis | 56 | 4.35(3.91,4.67) | 20.38(15.65,26.52) |
| Rheumatoid factor positive | 41 | 4.37(3.86,4.75) | 20.75(15.25,28.23) |
| Muscle injury | 44 | 4.34(3.84,4.70) | 20.23(15.03,27.24) |
| Helicobacter infection | 50 | 4.19(3.72,4.53) | 18.28(13.83,24.15) |
| Myositis | 40 | 4.22(3.69,4.60) | 18.65(13.65,25.46) |
| Knee arthroplasty | 84 | 4.03(3.67,4.29) | 16.33(13.17,20.26) |
| Anti-cyclic citrullinated peptide antibody positive | 41 | 4.18(3.66,4.55) | 18.10(13.31,24.63) |
| Walking aid user | 35 | 4.21(3.65,4.61) | 18.49(13.25,25.80) |
| Grip strength decreased | 44 | 4.12(3.62,4.48) | 17.42(12.94,23.44) |
| Hypercholesterolaemia | 36 | 4.17(3.61,4.56) | 17.96(12.93,24.95) |
| Joint range of motion decreased | 55 | 4.01(3.56,4.33) | 16.08(12.33,20.98) |
| Hip arthroplasty | 59 | 3.93(3.50,4.24) | 15.27(11.81,19.73) |
| Deep vein thrombosis postoperative | 14 | 4.07(3.17,4.69) | 16.81(9.92,28.49) |
| Wound infection | 35 | 3.73(3.17,4.13) | 13.28(9.52,18.52) |
| Pemphigus | 44 | 3.59(3.09,3.95) | 12.04(8.95,16.20) |
| Pericarditis | 48 | 3.54(3.06,3.88) | 11.62(8.75,15.44) |
| Spinal operation | 42 | 3.57(3.05,3.93) | 11.84(8.74,16.04) |
| Hand deformity | 45 | 3.55(3.05,3.90) | 11.67(8.71,15.65) |
| Back disorder | 47 | 3.53(3.05,3.88) | 11.55(8.67,15.39) |
| Breast cancer stage II | 36 | 3.53(2.98,3.93) | 11.57(8.33,16.06) |
| Irritable bowel syndrome | 50 | 3.36(2.89,3.69) | 10.24(7.76,13.53) |
| Synovitis | 50 | 3.23(2.76,3.56) | 9.37(7.09,12.37) |
| Exostosis | 24 | 3.42(2.74,3.90) | 10.71(7.17,16.01) |
| Blood cholesterol increased | 78 | 3.11(2.73,3.38) | 8.63(6.90,10.78) |
| Fibromyalgia | 52 | 3.17(2.71,3.50) | 9.02(6.87,11.85) |
| **PT** | **Report numbers** | **IC (95% CI)** | **ROR (95%CI)** |
| Pulmonary fibrosis | 40 | 3.21(2.69,3.59) | 9.27(6.80,12.66) |
| Impaired healing | 60 | 3.09(2.66,3.40) | 8.53(6.62,11.00) |
| Therapy interrupted | 130 | 2.91(2.62,3.12) | 7.50(6.31,8.92) |
| Glossodynia | 46 | 3.11(2.62,3.46) | 8.61(6.44,11.50) |
| Liver injury | 47 | 3.09(2.60,3.44) | 8.51(6.39,11.34) |
| Knee operation | 25 | 3.24(2.57,3.71) | 9.43(6.36,13.97) |
| Spinal fusion surgery | 14 | 3.41(2.51,4.03) | 10.65(6.30,18.02) |
| Surgery | 91 | 2.81(2.46,3.06) | 7.01(5.70,8.61) |
| Lupus vulgaris | 8 | 3.65(2.44,4.45) | 12.54(6.24,25.22) |
| Type 2 diabetes mellitus | 50 | 2.86(2.39,3.20) | 7.26(5.49,9.58) |
| Systemic lupus erythematosus | 59 | 2.82(2.39,3.13) | 7.06(5.47,9.12) |
| Psoriasis | 195 | 2.59(2.36,2.76) | 6.04(5.24,6.95) |
| Hip surgery | 14 | 3.07(2.17,3.69) | 8.39(4.96,14.19) |
| Limb operation | 13 | 3.06(2.12,3.71) | 8.36(4.85,14.41) |
| Wound | 44 | 2.62(2.12,2.98) | 6.16(4.58,8.29) |
| Disability | 35 | 2.65(2.09,3.05) | 6.28(4.51,8.76) |
| Dental operation | 9 | 3.21(2.07,3.97) | 9.26(4.81,17.84) |
| Intervertebral disc operation | 7 | 3.33(2.03,4.18) | 10.07(4.78,21.21) |
| Arthropathy | 73 | 2.41(2.02,2.69) | 5.31(4.22,6.68) |
| Shoulder arthroplasty | 9 | 3.08(1.94,3.84) | 8.44(4.39,16.26) |
| Gait inability | 50 | 2.40(1.93,2.73) | 5.27(3.99,6.95) |
| Anti-cyclic citrullinated peptide antibody | 6 | 3.33(1.91,4.24) | 10.05(4.49,22.50) |
| Delirium | 38 | 2.35(1.81,2.73) | 5.09(3.70,7.00) |
| Medical procedure | 7 | 3.08(1.77,3.93) | 8.44(4.01,17.74) |
| Rotator cuff syndrome | 17 | 2.53(1.71,3.10) | 5.77(3.59,9.30) |
| Autoimmune disorder | 15 | 2.57(1.70,3.17) | 5.95(3.58,9.88) |
| Musculoskeletal stiffness | 86 | 2.04(1.68,2.29) | 4.10(3.32,5.07) |
| Joint stiffness | 30 | 2.28(1.67,2.71) | 4.85(3.39,6.95) |
| Lower limb fracture | 23 | 2.36(1.66,2.85) | 5.13(3.41,7.73) |
| Eye injury | 8 | 2.87(1.66,3.67) | 7.32(3.65,14.65) |
| Sinusitis | 95 | 2.00(1.66,2.24) | 3.99(3.26,4.88) |
| Dactylitis | 6 | 3.07(1.65,3.98) | 8.38(3.75,18.71) |
| Hepatic enzyme increased | 63 | 2.07(1.65,2.37) | 4.19(3.27,5.37) |
| Lower respiratory tract infection | 49 | 2.12(1.64,2.46) | 4.34(3.28,5.75) |
| Ulcer haemorrhage | 11 | 2.66(1.64,3.36) | 6.32(3.50,11.43) |
| Facet joint syndrome | 7 | 2.92(1.61,3.77) | 7.54(3.59,15.86) |
| Injury | 53 | 2.06(1.60,2.38) | 4.16(3.17,5.44) |
| Illness | 96 | 1.94(1.60,2.18) | 3.82(3.13,4.68) |
| Foot operation | 10 | 2.67(1.59,3.39) | 6.36(3.42,11.83) |
| Oedema | 48 | 2.07(1.59,2.41) | 4.20(3.16,5.58) |
| Bursitis | 15 | 2.45(1.58,3.05) | 5.45(3.28,9.05) |
| **PT** | **Report numbers** | **IC (95% CI)** | **ROR (95%CI)** |
| Shoulder operation | 9 | 2.71(1.57,3.47) | 6.56(3.41,12.62) |
| Osteoarthritis | 40 | 2.08(1.56,2.46) | 4.23(3.10,5.78) |
| Post procedural infection | 13 | 2.48(1.54,3.12) | 5.58(3.24,9.62) |
| Intervertebral disc protrusion | 23 | 2.24(1.54,2.73) | 4.71(3.13,7.09) |
| Discomfort | 58 | 1.97(1.53,2.28) | 3.91(3.02,5.06) |
| Acne | 71 | 1.88(1.49,2.17) | 3.69(2.92,4.66) |
| Axial spondyloarthritis | 5 | 3.05(1.49,4.04) | 8.29(3.43,20.01) |
| Blister | 49 | 1.96(1.49,2.30) | 3.90(2.94,5.16) |
| Stomatitis | 55 | 1.92(1.47,2.24) | 3.78(2.90,4.92) |
| Joint arthroplasty | 6 | 2.86(1.45,3.77) | 7.27(3.26,16.22) |
| Heart valve incompetence | 7 | 2.75(1.44,3.60) | 6.71(3.19,14.10) |
| Foot deformity | 15 | 2.28(1.41,2.88) | 4.85(2.92,8.05) |
| Skin cancer | 25 | 2.07(1.41,2.55) | 4.21(2.84,6.24) |
| Joint swelling | 96 | 1.74(1.40,1.98) | 3.33(2.72,4.07) |
| Oral surgery | 6 | 2.71(1.29,3.62) | 6.54(2.93,14.57) |
| Ear infection | 25 | 1.93(1.26,2.40) | 3.81(2.57,5.64) |
| Arthralgia | 265 | 1.46(1.26,1.61) | 2.75(2.44,3.11) |
| Mobility decreased | 55 | 1.69(1.24,2.01) | 3.23(2.48,4.21) |
| Joint dislocation | 12 | 2.21(1.23,2.88) | 4.62(2.62,8.14) |
| Infection | 98 | 1.56(1.22,1.80) | 2.94(2.41,3.59) |
| Rheumatic disorder | 6 | 2.63(1.22,3.54) | 6.20(2.78,13.83) |
| Skin disorder | 29 | 1.84(1.22,2.27) | 3.57(2.48,5.14) |
| C-reactive protein abnormal | 10 | 2.29(1.21,3.02) | 4.89(2.63,9.10) |
| COVID-19 | 155 | 1.48(1.21,1.67) | 2.78(2.37,3.26) |
| Fluid retention | 41 | 1.73(1.21,2.10) | 3.31(2.43,4.49) |
| Inflammation | 40 | 1.73(1.21,2.11) | 3.32(2.43,4.53) |
| Therapeutic product effect incomplete | 62 | 1.61(1.19,1.91) | 3.05(2.37,3.91) |
| Oral herpes | 19 | 1.95(1.18,2.48) | 3.85(2.46,6.04) |
| Infusion related reaction | 48 | 1.65(1.17,2.00) | 3.15(2.37,4.18) |
| Neck surgery | 6 | 2.58(1.17,3.49) | 5.99(2.69,13.36) |
| Migraine | 65 | 1.58(1.17,1.87) | 2.98(2.34,3.81) |
| Peripheral venous disease | 7 | 2.44(1.13,3.29) | 5.41(2.58,11.37) |
| Amnesia | 41 | 1.65(1.13,2.02) | 3.13(2.30,4.26) |
| Swelling | 65 | 1.54(1.12,1.83) | 2.90(2.27,3.70) |
| Gastrointestinal disorder | 80 | 1.48(1.11,1.75) | 2.79(2.24,3.48) |
| Arthritis | 54 | 1.56(1.11,1.88) | 2.95(2.26,3.85) |
| Lipids increased | 5 | 2.65(1.08,3.63) | 6.26(2.60,15.08) |
| Nasopharyngitis | 115 | 1.39(1.08,1.61) | 2.61(2.18,3.14) |
| Wheezing | 41 | 1.58(1.06,1.96) | 3.00(2.21,4.08) |
| Meniscus injury | 9 | 2.18(1.04,2.94) | 4.52(2.35,8.70) |
| Local reaction | 4 | 2.76(1.00,3.84) | 6.79(2.54,18.17) |
| **PT** | **Report numbers** | **IC (95% CI)** | **ROR (95%CI)** |
| Respiratory syncytial virus infection | 10 | 2.07(1.00,2.80) | 4.21(2.27,7.84) |
| Rheumatoid arthritis | 71 | 1.37(0.97,1.65) | 2.58(2.04,3.26) |
| Rehabilitation therapy | 7 | 2.27(0.97,3.12) | 4.82(2.30,10.13) |
| Peripheral swelling | 109 | 1.28(0.96,1.50) | 2.42(2.01,2.92) |
| Ill-defined disorder | 44 | 1.45(0.95,1.81) | 2.74(2.04,3.68) |
| C-reactive protein | 4 | 2.71(0.94,3.79) | 6.54(2.44,17.49) |
| Cataract | 38 | 1.48(0.94,1.87) | 2.79(2.03,3.84) |
| Pyelocaliectasis | 4 | 2.70(0.94,3.78) | 6.52(2.44,17.43) |
| Coronavirus infection | 10 | 2.01(0.94,2.74) | 4.04(2.17,7.51) |
| Spinal stenosis | 7 | 2.23(0.93,3.08) | 4.70(2.24,9.86) |
| Sleep disorder | 44 | 1.43(0.92,1.78) | 2.69(2.00,3.61) |
| SARS-CoV-2 test positive | 16 | 1.76(0.92,2.35) | 3.40(2.08,5.55) |
| Contusion | 56 | 1.36(0.92,1.68) | 2.57(1.98,3.35) |
| Dyspepsia | 52 | 1.34(0.88,1.67) | 2.54(1.93,3.34) |
| Pain in extremity | 146 | 1.14(0.87,1.34) | 2.20(1.87,2.59) |
| Device loosening | 4 | 2.61(0.84,3.69) | 6.09(2.28,16.28) |
| Joint injury | 16 | 1.68(0.84,2.26) | 3.20(1.96,5.23) |
| Chest pain | 82 | 1.19(0.82,1.45) | 2.28(1.84,2.84) |
| Neck pain | 33 | 1.40(0.82,1.81) | 2.64(1.88,3.72) |
| Prostatic operation | 4 | 2.58(0.82,3.66) | 5.99(2.24,16.00) |
| Porphyria acute | 4 | 2.57(0.81,3.65) | 5.96(2.23,15.92) |
| Seronegative arthritis | 4 | 2.57(0.81,3.65) | 5.95(2.23,15.89) |
| Herpes zoster | 35 | 1.36(0.80,1.76) | 2.56(1.84,3.57) |
| Inflammatory marker increased | 7 | 2.08(0.78,2.94) | 4.24(2.02,8.90) |
| Colectomy | 7 | 2.08(0.78,2.93) | 4.23(2.01,8.88) |
| Retinitis | 4 | 2.54(0.78,3.62) | 5.82(2.18,15.54) |
| Hysterectomy | 8 | 1.97(0.76,2.77) | 3.91(1.95,7.83) |
| General physical health deterioration | 56 | 1.19(0.75,1.51) | 2.28(1.76,2.97) |
| Hypoaesthesia | 70 | 1.13(0.74,1.42) | 2.20(1.74,2.78) |
| Musculoskeletal disorder | 15 | 1.61(0.74,2.21) | 3.05(1.84,5.06) |
| Nerve compression | 9 | 1.87(0.74,2.64) | 3.67(1.91,7.05) |
| Skin plaque | 9 | 1.87(0.73,2.64) | 3.67(1.91,7.05) |
| Ligament rupture | 6 | 2.15(0.73,3.06) | 4.43(1.99,9.87) |
| Breast cancer stage III | 8 | 1.94(0.72,2.74) | 3.82(1.91,7.65) |
| Wrist fracture | 10 | 1.78(0.71,2.51) | 3.44(1.85,6.40) |
| Upper respiratory tract infection | 27 | 1.34(0.69,1.79) | 2.52(1.73,3.68) |
| Carpal tunnel syndrome | 11 | 1.72(0.69,2.41) | 3.29(1.82,5.94) |
| Tendonitis | 11 | 1.67(0.65,2.37) | 3.19(1.77,5.77) |
| Pain | 264 | 0.85(0.64,1.00) | 1.80(1.59,2.03) |
| Retinal artery thrombosis | 3 | 2.71(0.64,3.92) | 6.55(2.08,20.69) |
| **PT** | **Report numbers** | **IC (95% CI)** | **ROR (95%CI)** |
| Therapeutic product effect decreased | 30 | 1.24(0.64,1.68) | 2.37(1.66,3.39) |
| Sleeve gastrectomy | 3 | 2.69(0.62,3.90) | 6.46(2.05,20.31) |
| Limb deformity | 4 | 2.35(0.59,3.43) | 5.12(1.92,13.66) |
| Post procedural complication | 11 | 1.57(0.55,2.27) | 2.98(1.65,5.38) |
| Nephrolithiasis | 24 | 1.21(0.52,1.69) | 2.31(1.55,3.44) |
| Back pain | 92 | 0.82(0.47,1.07) | 1.77(1.44,2.17) |
| Kidney infection | 13 | 1.41(0.47,2.05) | 2.65(1.54,4.57) |
| Spondylitis | 5 | 2.03(0.47,3.01) | 4.08(1.70,9.82) |
| Loss of personal independence in daily activities | 29 | 1.08(0.46,1.52) | 2.12(1.47,3.05) |
| Foot fracture | 12 | 1.43(0.45,2.10) | 2.70(1.53,4.76) |
| Gastrointestinal erosion | 3 | 2.51(0.44,3.72) | 5.71(1.83,17.81) |
| Prescribed overdose | 13 | 1.38(0.44,2.02) | 2.60(1.51,4.47) |
| Respiratory tract infection | 15 | 1.27(0.39,1.87) | 2.40(1.45,3.99) |
| Cholecystectomy | 7 | 1.68(0.37,2.53) | 3.19(1.52,6.71) |
| Laryngitis | 8 | 1.59(0.37,2.39) | 3.00(1.50,6.00) |
| Sacral pain | 3 | 2.44(0.37,3.64) | 5.42(1.74,16.88) |
| Abdominal pain upper | 75 | 0.74(0.36,1.02) | 1.68(1.34,2.10) |
| Hypertension | 75 | 0.74(0.35,1.01) | 1.67(1.33,2.09) |
| Dermal cyst | 4 | 2.11(0.34,3.19) | 4.32(1.62,11.52) |
| Gait disturbance | 72 | 0.72(0.33,1.00) | 1.65(1.31,2.08) |
| Staphylococcal infection | 15 | 1.20(0.33,1.80) | 2.30(1.38,3.81) |
| Unevaluable event | 34 | 0.90(0.33,1.30) | 1.86(1.33,2.61) |
| Influenza | 46 | 0.79(0.30,1.14) | 1.73(1.30,2.32) |
| Oophorectomy | 3 | 2.37(0.30,3.58) | 5.17(1.66,16.10) |
| Pharyngitis streptococcal | 8 | 1.51(0.30,2.31) | 2.85(1.42,5.70) |
| Bronchitis | 31 | 0.89(0.30,1.32) | 1.86(1.31,2.65) |
| Atrioventricular block | 6 | 1.70(0.29,2.61) | 3.26(1.46,7.25) |
| Guttate psoriasis | 3 | 2.36(0.29,3.56) | 5.12(1.65,15.93) |
| Memory impairment | 54 | 0.73(0.28,1.05) | 1.66(1.27,2.17) |
| Pulmonary thrombosis | 8 | 1.47(0.25,2.27) | 2.76(1.38,5.53) |
| Immune system disorder | 9 | 1.39(0.25,2.15) | 2.62(1.36,5.04) |
| Spinal pain | 9 | 1.38(0.24,2.14) | 2.60(1.35,4.99) |
| Bone disorder | 9 | 1.35(0.21,2.11) | 2.55(1.33,4.91) |
| Drug tolerance decreased | 4 | 1.97(0.21,3.05) | 3.92(1.47,10.47) |
| Tooth abscess | 7 | 1.50(0.20,2.35) | 2.83(1.35,5.94) |
| Road traffic accident | 17 | 1.01(0.20,1.58) | 2.02(1.25,3.25) |
| Liver disorder | 18 | 0.96(0.17,1.51) | 1.95(1.23,3.09) |
| Fall | 102 | 0.49(0.16,0.73) | 1.41(1.16,1.71) |
| Vitreous floaters | 7 | 1.46(0.16,2.32) | 2.76(1.31,5.79) |
| Laboratory test abnormal | 16 | 0.99(0.15,1.58) | 1.99(1.22,3.25) |
| **PT** | **Report numbers** | **IC (95% CI)** | **ROR (95%CI)** |
| Bladder operation | 3 | 2.22(0.15,3.43) | 4.66(1.50,14.47) |
| Viral infection | 15 | 1.02(0.15,1.62) | 2.03(1.22,3.36) |
| Urinary tract infection | 58 | 0.58(0.15,0.89) | 1.50(1.16,1.94) |
| Myelofibrosis | 4 | 1.91(0.14,2.99) | 3.76(1.41,10.02) |
| Procedural pain | 13 | 1.07(0.14,1.72) | 2.10(1.22,3.63) |
| Tendon pain | 6 | 1.54(0.13,2.45) | 2.91(1.31,6.48) |
| Respiratory tract congestion | 9 | 1.26(0.12,2.03) | 2.40(1.25,4.62) |
| Abdominal discomfort | 60 | 0.55(0.12,0.86) | 1.47(1.14,1.89) |
| Weight increased | 69 | 0.51(0.11,0.80) | 1.43(1.13,1.81) |
| Pyrexia | 100 | 0.44(0.11,0.68) | 1.36(1.12,1.66) |
| Pneumothorax | 9 | 1.25(0.11,2.01) | 2.38(1.24,4.57) |
| Plantar fasciitis | 4 | 1.87(0.10,2.95) | 3.66(1.37,9.75) |
| Lupus-like syndrome | 6 | 1.52(0.10,2.43) | 2.87(1.29,6.38) |
| Shoulder fracture | 3 | 2.17(0.10,3.38) | 4.50(1.45,13.98) |
| Intentional dose omission | 13 | 1.03(0.10,1.68) | 2.05(1.19,3.53) |
| Tendon rupture | 7 | 1.39(0.09,2.25) | 2.63(1.25,5.52) |
| Sciatica | 9 | 1.22(0.08,1.98) | 2.33(1.21,4.49) |
| Gastric haemorrhage | 7 | 1.39(0.08,2.24) | 2.61(1.24,5.48) |
| Urticaria | 51 | 0.55(0.08,0.88) | 1.46(1.11,1.92) |
| Ligament sprain | 7 | 1.38(0.08,2.23) | 2.60(1.24,5.46) |
| Cardiac operation | 6 | 1.49(0.08,2.40) | 2.81(1.26,6.26) |
| Pneumonia | 96 | 0.41(0.07,0.65) | 1.33(1.09,1.62) |
| Diverticulitis | 13 | 1.00(0.06,1.64) | 2.00(1.16,3.45) |
| Oesophageal candidiasis | 4 | 1.82(0.05,2.90) | 3.52(1.32,9.40) |
| Candida infection | 10 | 1.12(0.04,1.84) | 2.17(1.17,4.03) |
| Drug hypersensitivity | 68 | 0.42(0.01,0.71) | 1.34(1.05,1.69) |
| Thrombocytosis | 4 | 1.78(0.01,2.86) | 3.43(1.28,9.14) |
| Bone erosion | 4 | 1.76(0.00,2.84) | 3.40(1.27,9.06) |
| Confusional state | 47 | 0.48(0.00,0.83) | 1.40(1.05,1.86) |
| Thrombosis | 27 | 0.64(0.00,1.10) | 1.56(1.07,2.28) |

Table S4 All positive adverse events of tofacitinib at the PT level

| **PT** | **Report numbers** | **IC (95% CI)** | **ROR (95%CI)** |
| --- | --- | --- | --- |
| Swollen joint count increased | 976 | 6.93(6.82,7.00) | 121.71(111.35,133.04) |
| Rheumatic fever | 1168 | 6.85(6.75,6.92) | 115.01(106.31,124.43) |
| Facet joint syndrome | 536 | 6.50(6.36,6.60) | 90.37(81.14,100.65) |
| Deep vein thrombosis postoperative | 407 | 6.50(6.34,6.62) | 90.81(80.14,102.89) |
| C-reactive protein | 240 | 6.55(6.33,6.70) | 93.54(78.89,110.91) |
| X-ray abnormal | 280 | 6.13(5.94,6.28) | 70.18(60.95,80.81) |
| Lupus vulgaris | 177 | 6.18(5.93,6.36) | 72.30(60.22,86.80) |
| Duodenal ulcer perforation | 1129 | 6.00(5.91,6.08) | 64.17(59.97,68.67) |
| Rheumatoid factor positive | 1079 | 5.99(5.89,6.06) | 63.49(59.24,68.04) |
| Anti-cyclic citrullinated peptide antibody positive | 1223 | 5.92(5.82,5.99) | 60.52(56.74,64.55) |
| Adjustment disorder with depressed mood | 296 | 5.87(5.68,6.01) | 58.65(51.41,66.91) |
| Dislocation of vertebra | 266 | 5.76(5.56,5.91) | 54.29(47.33,62.28) |
| Helicobacter infection | 1144 | 5.49(5.40,5.57) | 45.07(42.28,48.06) |
| Anti-cyclic citrullinated peptide antibody | 109 | 5.69(5.38,5.92) | 51.77(41.61,64.41) |
| Retinitis | 168 | 5.59(5.33,5.77) | 48.01(40.47,56.95) |
| Pemphigus | 1491 | 5.39(5.30,5.45) | 41.85(39.58,44.25) |
| Hand deformity | 1531 | 5.34(5.25,5.40) | 40.44(38.29,42.72) |
| Muscle injury | 736 | 5.26(5.14,5.35) | 38.38(35.48,41.52) |
| Peripheral venous disease | 399 | 5.29(5.12,5.41) | 39.01(35.05,43.42) |
| Folliculitis | 947 | 5.22(5.11,5.29) | 37.16(34.68,39.82) |
| Sleep disorder due to general medical condition, insomnia type | 583 | 5.13(4.99,5.23) | 34.97(32.04,38.17) |
| Pericarditis | 1343 | 5.04(4.95,5.10) | 32.85(31.01,34.80) |
| Subcutaneous drug absorption impaired | 58 | 5.27(4.84,5.59) | 38.68(28.89,51.78) |
| Synovitis | 1605 | 4.89(4.80,4.95) | 29.56(28.06,31.15) |
| C-reactive protein abnormal | 550 | 4.89(4.75,4.99) | 29.60(27.08,32.36) |
| Glossodynia | 1542 | 4.82(4.74,4.89) | 28.34(26.87,29.89) |
| Lip dry | 550 | 4.88(4.74,4.98) | 29.39(26.89,32.13) |
| Grip strength | 34 | 5.23(4.66,5.63) | 37.45(24.97,56.17) |
| Swollen joint count | 32 | 4.98(4.39,5.40) | 31.57(21.25,46.90) |
| Ankle arthroplasty | 44 | 4.87(4.37,5.23) | 29.28(21.18,40.47) |
| Bone erosion | 219 | 4.52(4.30,4.68) | 22.93(19.95,26.35) |
| Breast cancer stage III | 398 | 4.36(4.19,4.48) | 20.49(18.50,22.71) |
| Live birth | 459 | 4.34(4.18,4.45) | 20.21(18.37,22.24) |
| Bursitis | 522 | 4.31(4.17,4.42) | 19.89(18.18,21.75) |
| Wound | 1400 | 4.23(4.15,4.30) | 18.82(17.82,19.88) |
| Hypercholesterolaemia | 345 | 4.32(4.14,4.44) | 19.91(17.84,22.23) |
| **PT** | **Report numbers** | **IC (95% CI)** | **ROR (95%CI)** |
| Systemic lupus erythematosus | 1593 | 4.18(4.10,4.24) | 18.17(17.27,19.13) |
| Joint range of motion decreased | 608 | 4.21(4.07,4.30) | 18.46(17.00,20.06) |
| Irritable bowel syndrome | 886 | 4.17(4.06,4.25) | 18.01(16.82,19.29) |
| Fibromyalgia | 1047 | 4.15(4.05,4.22) | 17.75(16.66,18.90) |
| Wound infection | 442 | 4.18(4.02,4.29) | 18.13(16.45,19.97) |
| Type 2 diabetes mellitus | 1229 | 4.10(4.01,4.17) | 17.18(16.21,18.21) |
| Joint destruction | 80 | 4.37(4.00,4.63) | 20.64(16.40,25.97) |
| Porphyria acute | 69 | 4.39(3.99,4.68) | 21.00(16.39,26.92) |
| Seronegative arthritis | 68 | 4.36(3.96,4.65) | 20.60(16.05,26.45) |
| Drug tolerance decreased | 143 | 4.24(3.96,4.44) | 18.85(15.89,22.36) |
| Autoimmune disorder | 408 | 4.11(3.95,4.23) | 17.27(15.61,19.09) |
| Joint ankylosis | 45 | 4.40(3.90,4.75) | 21.09(15.48,28.72) |
| Grip strength decreased | 386 | 4.06(3.90,4.19) | 16.73(15.09,18.55) |
| Liver injury | 904 | 4.00(3.89,4.08) | 16.04(15.00,17.16) |
| Blepharospasm | 227 | 4.10(3.88,4.26) | 17.12(14.96,19.60) |
| Onychomadesis | 164 | 4.11(3.86,4.30) | 17.31(14.77,20.29) |
| Sciatica | 561 | 3.82(3.68,3.92) | 14.12(12.96,15.37) |
| Contraindicated product administered | 1318 | 3.75(3.66,3.82) | 13.44(12.72,14.21) |
| Blood cholesterol increased | 1275 | 3.75(3.65,3.81) | 13.41(12.67,14.19) |
| Red blood cell sedimentation rate abnormal | 121 | 3.87(3.57,4.09) | 14.63(12.17,17.58) |
| Hip arthroplasty | 495 | 3.71(3.56,3.82) | 13.08(11.95,14.32) |
| Psoriatic arthropathy | 1339 | 3.65(3.56,3.71) | 12.53(11.86,13.24) |
| Impaired healing | 900 | 3.62(3.51,3.70) | 12.34(11.54,13.19) |
| Obesity | 463 | 3.61(3.45,3.72) | 12.17(11.09,13.37) |
| Sleep disorder due to general medical condition, hypersomnia type | 14 | 4.32(3.42,4.94) | 20.02(10.97,36.54) |
| Pulmonary fibrosis | 494 | 3.52(3.37,3.63) | 11.49(10.50,12.58) |
| Arthropathy | 1461 | 3.31(3.22,3.37) | 9.92(9.41,10.45) |
| Knee arthroplasty | 495 | 3.26(3.11,3.36) | 9.55(8.73,10.45) |
| Nail disorder | 210 | 3.32(3.09,3.48) | 9.98(8.69,11.46) |
| Onychomycosis | 109 | 3.40(3.09,3.63) | 10.59(8.74,12.83) |
| Tender joint count | 12 | 4.05(3.07,4.72) | 16.56(8.86,30.95) |
| Prescribed overdose | 483 | 3.21(3.06,3.32) | 9.26(8.46,10.14) |
| Hepatic enzyme increased | 1378 | 3.09(3.00,3.16) | 8.52(8.08,9.00) |
| Exostosis | 186 | 3.21(2.97,3.39) | 9.25(7.99,10.71) |
| Walking aid user | 151 | 3.23(2.96,3.43) | 9.40(7.99,11.06) |
| Hyperadrenocorticism | 16 | 3.79(2.95,4.37) | 13.85(8.29,23.14) |
| Infusion related reaction | 1323 | 3.01(2.92,3.07) | 8.04(7.61,8.50) |
| Lower limb fracture | 380 | 3.07(2.90,3.19) | 8.39(7.58,9.30) |
| **PT** | **Report numbers** | **IC (95% CI)** | **ROR (95%CI)** |
| Maternal exposure timing unspecified | 70 | 3.23(2.83,3.51) | 9.37(7.38,11.90) |
| Foot deformity | 247 | 3.04(2.83,3.19) | 8.21(7.23,9.32) |
| Bone cyst | 29 | 3.44(2.82,3.88) | 10.89(7.49,15.82) |
| Premature ageing | 15 | 3.69(2.82,4.29) | 12.88(7.60,21.85) |
| Parathyroid disorder | 26 | 3.47(2.82,3.93) | 11.09(7.47,16.46) |
| Carbon dioxide abnormal | 16 | 3.64(2.80,4.22) | 12.47(7.49,20.76) |
| Ulcer haemorrhage | 123 | 3.04(2.74,3.26) | 8.23(6.87,9.85) |
| Discomfort | 1123 | 2.82(2.72,2.89) | 7.04(6.63,7.47) |
| Eye injury | 66 | 3.10(2.69,3.39) | 8.55(6.69,10.93) |
| Red blood cell sedimentation rate increased | 197 | 2.88(2.65,3.05) | 7.37(6.40,8.50) |
| Infusion site reaction | 53 | 3.10(2.65,3.43) | 8.59(6.53,11.30) |
| Swelling | 1607 | 2.72(2.64,2.78) | 6.60(6.28,6.94) |
| Blister | 902 | 2.74(2.63,2.82) | 6.70(6.27,7.16) |
| Lupus-like syndrome | 149 | 2.91(2.63,3.10) | 7.49(6.37,8.82) |
| Osteoarthritis | 671 | 2.74(2.62,2.84) | 6.70(6.20,7.23) |
| Mobility decreased | 1188 | 2.69(2.59,2.76) | 6.45(6.09,6.84) |
| Injury | 881 | 2.69(2.58,2.77) | 6.45(6.04,6.90) |
| Hypocholesterolaemia | 9 | 3.71(2.57,4.48) | 13.12(6.44,26.71) |
| Finger deformity | 95 | 2.91(2.57,3.15) | 7.50(6.12,9.21) |
| Musculoskeletal stiffness | 1388 | 2.61(2.52,2.68) | 6.11(5.79,6.45) |
| Night sweats | 484 | 2.65(2.50,2.76) | 6.28(5.73,6.87) |
| Pustular psoriasis | 58 | 2.93(2.49,3.24) | 7.62(5.87,9.90) |
| Rheumatoid arthritis | 1765 | 2.56(2.48,2.62) | 5.89(5.62,6.18) |
| Laryngitis | 173 | 2.73(2.48,2.91) | 6.63(5.70,7.71) |
| Drug tolerance | 93 | 2.81(2.47,3.06) | 7.01(5.70,8.61) |
| Gait inability | 601 | 2.58(2.45,2.68) | 5.99(5.52,6.49) |
| C-reactive protein increased | 521 | 2.56(2.42,2.67) | 5.92(5.42,6.45) |
| Lower respiratory tract infection | 699 | 2.54(2.41,2.63) | 5.81(5.39,6.27) |
| Road traffic accident | 526 | 2.54(2.40,2.65) | 5.82(5.34,6.35) |
| Polyarthritis | 88 | 2.75(2.40,3.00) | 6.73(5.44,8.32) |
| Joint dislocation | 154 | 2.64(2.38,2.83) | 6.24(5.32,7.32) |
| Drug intolerance | 1532 | 2.46(2.38,2.52) | 5.50(5.23,5.79) |
| Stomatitis | 872 | 2.48(2.37,2.56) | 5.57(5.21,5.96) |
| Joint swelling | 1708 | 2.45(2.37,2.50) | 5.45(5.19,5.72) |
| Loose body in joint | 11 | 3.38(2.36,4.08) | 10.43(5.65,19.25) |
| Ear injury | 16 | 3.20(2.36,3.78) | 9.18(5.55,15.16) |
| Rheumatoid factor negative | 11 | 3.32(2.29,4.01) | 9.97(5.41,18.38) |
| Oedema | 652 | 2.42(2.29,2.51) | 5.35(4.95,5.78) |
| Wheezing | 781 | 2.41(2.29,2.49) | 5.31(4.95,5.70) |
| **PT** | **Report numbers** | **IC (95% CI)** | **ROR (95%CI)** |
| General physical health deterioration | 1387 | 2.38(2.29,2.44) | 5.19(4.92,5.48) |
| Therapeutic product effect decreased | 1266 | 2.34(2.25,2.41) | 5.07(4.79,5.36) |
| Discouragement | 50 | 2.71(2.24,3.05) | 6.55(4.95,8.68) |
| Product label confusion | 97 | 2.56(2.22,2.80) | 5.88(4.81,7.19) |
| Back injury | 142 | 2.49(2.21,2.69) | 5.62(4.76,6.64) |
| Inflammation | 645 | 2.32(2.19,2.42) | 5.00(4.62,5.40) |
| Liver disorder | 501 | 2.33(2.19,2.44) | 5.04(4.61,5.50) |
| Disease risk factor | 13 | 3.12(2.18,3.76) | 8.70(4.99,15.19) |
| Maternal exposure during pregnancy | 1160 | 2.26(2.16,2.33) | 4.78(4.51,5.07) |
| Treatment failure | 1216 | 2.26(2.16,2.32) | 4.77(4.51,5.05) |
| Osteolysis | 29 | 2.77(2.15,3.21) | 6.83(4.72,9.88) |
| Coeliac disease | 118 | 2.44(2.13,2.66) | 5.42(4.52,6.50) |
| Alanine aminotransferase abnormal | 35 | 2.63(2.07,3.03) | 6.20(4.43,8.67) |
| Hepatic enzyme | 6 | 3.47(2.06,4.39) | 11.11(4.35,28.40) |
| Pregnancy | 170 | 2.30(2.05,2.48) | 4.93(4.23,5.73) |
| Hepatic cirrhosis | 208 | 2.25(2.03,2.42) | 4.77(4.16,5.47) |
| Breast cancer stage II | 143 | 2.27(2.00,2.47) | 4.83(4.09,5.70) |
| Rash vesicular | 61 | 2.41(1.98,2.71) | 5.31(4.12,6.84) |
| Prescribed underdose | 277 | 2.16(1.96,2.31) | 4.48(3.97,5.04) |
| Lung disorder | 509 | 2.11(1.96,2.21) | 4.31(3.95,4.71) |
| Spondylitis | 50 | 2.36(1.89,2.70) | 5.15(3.89,6.81) |
| Spinal fusion surgery | 50 | 2.34(1.87,2.68) | 5.06(3.83,6.70) |
| Ill-defined disorder | 691 | 1.99(1.87,2.08) | 3.98(3.69,4.29) |
| Nail operation | 11 | 2.86(1.84,3.56) | 7.27(3.98,13.29) |
| Sleep disorder | 676 | 1.93(1.81,2.02) | 3.82(3.54,4.12) |
| Abdominal discomfort | 1637 | 1.87(1.79,1.93) | 3.65(3.47,3.83) |
| Liver function test increased | 259 | 1.97(1.77,2.12) | 3.93(3.47,4.44) |
| Delirium | 302 | 1.95(1.76,2.09) | 3.87(3.45,4.33) |
| Carbon dioxide decreased | 16 | 2.60(1.76,3.18) | 6.06(3.69,9.97) |
| Joint laxity | 10 | 2.81(1.73,3.54) | 7.01(3.73,13.20) |
| Musculoskeletal pain | 457 | 1.88(1.73,1.99) | 3.68(3.36,4.04) |
| Dry mouth | 663 | 1.84(1.71,1.93) | 3.57(3.31,3.86) |
| Exposure during pregnancy | 637 | 1.82(1.69,1.92) | 3.53(3.27,3.82) |
| Taste disorder | 213 | 1.90(1.68,2.07) | 3.74(3.27,4.28) |
| Psoriasis area severity index increased | 7 | 2.91(1.61,3.77) | 7.54(3.52,16.16) |
| Rheumatoid factor | 6 | 3.01(1.59,3.92) | 8.03(3.50,18.46) |
| Sinusitis | 837 | 1.70(1.58,1.78) | 3.24(3.03,3.47) |
| Sensitivity to weather change | 65 | 1.96(1.55,2.26) | 3.90(3.05,4.98) |
| **PT** | **Report numbers** | **IC (95% CI)** | **ROR (95%CI)** |
| Multiple-drug resistance | 39 | 2.07(1.53,2.45) | 4.19(3.05,5.75) |
| Intentional product use issue | 884 | 1.64(1.53,1.72) | 3.11(2.91,3.33) |
| Therapy non-responder | 399 | 1.67(1.51,1.79) | 3.19(2.89,3.52) |
| Joint stiffness | 212 | 1.73(1.50,1.89) | 3.31(2.89,3.80) |
| Hypersensitivity | 1363 | 1.57(1.48,1.63) | 2.96(2.81,3.12) |
| Epilepsy | 229 | 1.69(1.48,1.85) | 3.24(2.84,3.69) |
| Thyroxine decreased | 10 | 2.55(1.47,3.27) | 5.84(3.11,10.96) |
| Hypertension | 1431 | 1.54(1.45,1.60) | 2.90(2.75,3.06) |
| Infection | 1058 | 1.54(1.44,1.62) | 2.92(2.74,3.10) |
| Alopecia | 1594 | 1.52(1.43,1.58) | 2.86(2.72,3.01) |
| Gilbert's syndrome | 8 | 2.63(1.41,3.43) | 6.17(3.05,12.50) |
| Hypoaesthesia | 991 | 1.51(1.41,1.59) | 2.85(2.67,3.03) |
| Foot operation | 48 | 1.88(1.40,2.22) | 3.68(2.77,4.90) |
| General symptom | 53 | 1.85(1.40,2.18) | 3.61(2.75,4.74) |
| Wheelchair user | 42 | 1.91(1.39,2.27) | 3.75(2.77,5.09) |
| Hepatic fibrosis | 29 | 1.99(1.37,2.43) | 3.98(2.76,5.75) |
| Incorrect route of product administration | 155 | 1.63(1.37,1.83) | 3.10(2.65,3.64) |
| Fracture delayed union | 10 | 2.42(1.34,3.14) | 5.35(2.85,10.02) |
| Dactylitis | 15 | 2.13(1.26,2.73) | 4.39(2.63,7.31) |
| Ear infection | 191 | 1.48(1.24,1.65) | 2.79(2.42,3.22) |
| Contusion | 611 | 1.37(1.24,1.47) | 2.59(2.39,2.80) |
| Granuloma skin | 13 | 2.16(1.23,2.81) | 4.48(2.59,7.76) |
| Myositis | 59 | 1.66(1.22,1.97) | 3.15(2.44,4.07) |
| Parathyroidectomy | 8 | 2.40(1.19,3.21) | 5.30(2.62,10.70) |
| Gastrointestinal disorder | 768 | 1.30(1.18,1.39) | 2.46(2.29,2.65) |
| Onycholysis | 17 | 1.97(1.16,2.54) | 3.93(2.43,6.34) |
| Condition aggravated | 1845 | 1.23(1.16,1.29) | 2.35(2.24,2.46) |
| Skin injury | 30 | 1.76(1.15,2.19) | 3.38(2.36,4.85) |
| Dyspepsia | 541 | 1.28(1.14,1.39) | 2.44(2.24,2.65) |
| Drug-induced liver injury | 216 | 1.36(1.13,1.52) | 2.56(2.24,2.93) |
| Confusional state | 852 | 1.21(1.10,1.29) | 2.31(2.16,2.47) |
| Lipids increased | 17 | 1.91(1.09,2.48) | 3.76(2.33,6.07) |
| Synovial disorder | 8 | 2.30(1.09,3.11) | 4.94(2.45,9.97) |
| Urticaria | 877 | 1.20(1.09,1.28) | 2.29(2.15,2.45) |
| Disability | 143 | 1.33(1.05,1.53) | 2.51(2.13,2.96) |
| Adrenal insufficiency | 81 | 1.42(1.05,1.69) | 2.68(2.15,3.33) |
| Noninfective gingivitis | 17 | 1.82(1.01,2.39) | 3.54(2.19,5.72) |
| Amnesia | 314 | 1.16(0.97,1.29) | 2.23(2.00,2.50) |
| Adverse reaction | 94 | 1.31(0.97,1.55) | 2.48(2.02,3.03) |
| Immunodeficiency | 99 | 1.29(0.95,1.53) | 2.44(2.00,2.97) |
| Migraine | 501 | 1.08(0.94,1.19) | 2.12(1.94,2.32) |
| **PT** | **Report numbers** | **IC (95% CI)** | **ROR (95%CI)** |
| Nodule | 83 | 1.30(0.93,1.56) | 2.46(1.98,3.05) |
| Joint arthroplasty | 16 | 1.77(0.93,2.35) | 3.41(2.08,5.59) |
| Product use issue | 1216 | 1.00(0.91,1.07) | 2.01(1.90,2.12) |
| Drug hypersensitivity | 1122 | 1.01(0.91,1.08) | 2.01(1.89,2.13) |
| Nasopharyngitis | 965 | 1.01(0.90,1.08) | 2.01(1.88,2.14) |
| Therapeutic product effect incomplete | 697 | 1.01(0.89,1.11) | 2.02(1.87,2.18) |
| Tenosynovitis stenosans | 7 | 2.18(0.88,3.03) | 4.53(2.14,9.59) |
| Ear pain | 106 | 1.17(0.85,1.40) | 2.25(1.86,2.72) |
| Sacroiliitis | 17 | 1.64(0.82,2.20) | 3.11(1.93,5.02) |
| Adverse drug reaction | 480 | 0.96(0.81,1.07) | 1.95(1.78,2.13) |
| Peripheral swelling | 928 | 0.91(0.81,0.99) | 1.89(1.77,2.01) |
| Tonsillar disorder | 10 | 1.87(0.79,2.59) | 3.65(1.95,6.81) |
| Arthritis | 382 | 0.95(0.78,1.07) | 1.93(1.74,2.13) |
| Osteoporosis | 205 | 0.98(0.75,1.15) | 1.97(1.72,2.26) |
| Enthesopathy | 16 | 1.58(0.74,2.17) | 3.00(1.83,4.91) |
| Weight increased | 949 | 0.84(0.73,0.92) | 1.79(1.68,1.91) |
| Dermatitis psoriasiform | 16 | 1.57(0.73,2.15) | 2.97(1.81,4.86) |
| Medication error | 196 | 0.96(0.73,1.13) | 1.95(1.69,2.24) |
| Loss of personal independence in daily activities | 326 | 0.90(0.72,1.04) | 1.87(1.68,2.09) |
| Synovial fluid analysis | 4 | 2.48(0.72,3.56) | 5.59(2.04,15.28) |
| Rheumatoid nodule | 22 | 1.43(0.71,1.93) | 2.69(1.76,4.09) |
| Rheumatoid lung | 10 | 1.78(0.71,2.51) | 3.44(1.84,6.43) |
| Arthralgia | 1800 | 0.77(0.69,0.83) | 1.71(1.63,1.79) |
| Blood parathyroid hormone decreased | 14 | 1.56(0.66,2.19) | 2.96(1.75,5.01) |
| Abdominal pain upper | 839 | 0.78(0.66,0.86) | 1.71(1.60,1.83) |
| Hepatitis | 105 | 0.97(0.65,1.20) | 1.96(1.62,2.38) |
| Mucosal dryness | 14 | 1.51(0.61,2.13) | 2.85(1.68,4.82) |
| Tuberculin test positive | 16 | 1.45(0.60,2.03) | 2.73(1.67,4.46) |
| Respiratory disorder | 128 | 0.89(0.59,1.10) | 1.85(1.55,2.20) |
| Back disorder | 75 | 0.91(0.53,1.18) | 1.88(1.50,2.36) |
| Memory impairment | 555 | 0.64(0.50,0.74) | 1.56(1.43,1.69) |
| Asthma | 425 | 0.66(0.50,0.77) | 1.58(1.43,1.73) |
| Rash | 1577 | 0.58(0.49,0.64) | 1.49(1.42,1.57) |
| Gastroenteritis viral | 82 | 0.86(0.49,1.12) | 1.81(1.46,2.25) |
| Hepatic steatosis | 72 | 0.87(0.48,1.15) | 1.83(1.45,2.31) |
| Product dose omission in error | 108 | 0.80(0.48,1.03) | 1.74(1.44,2.10) |
| Gastrooesophageal reflux disease | 276 | 0.63(0.43,0.77) | 1.54(1.37,1.74) |
| Death neonatal | 9 | 1.57(0.43,2.33) | 2.96(1.53,5.71) |
| Muscular weakness | 392 | 0.58(0.42,0.70) | 1.50(1.36,1.66) |
| **PT** | **Report numbers** | **IC (95% CI)** | **ROR (95%CI)** |
| Pain | 2251 | 0.48(0.41,0.53) | 1.40(1.34,1.46) |
| Lip squamous cell carcinoma | 4 | 2.18(0.41,3.26) | 4.52(1.67,12.25) |
| Nail psoriasis | 7 | 1.70(0.40,2.55) | 3.25(1.54,6.87) |
| Upper respiratory tract infection | 174 | 0.60(0.35,0.79) | 1.52(1.31,1.76) |
| Chest pain | 529 | 0.43(0.29,0.54) | 1.35(1.24,1.47) |
| Blood cholesterol | 5 | 1.84(0.27,2.82) | 3.57(1.47,8.66) |
| Limb deformity | 11 | 1.30(0.27,1.99) | 2.46(1.36,4.45) |
| Upper respiratory tract infection bacterial | 4 | 2.03(0.27,3.11) | 4.09(1.52,11.05) |
| Neck pain | 190 | 0.50(0.26,0.68) | 1.42(1.23,1.63) |
| Cutaneous symptom | 7 | 1.56(0.26,2.41) | 2.94(1.40,6.21) |
| Decreased appetite | 756 | 0.36(0.24,0.45) | 1.28(1.20,1.38) |
| Rheumatoid factor increased | 8 | 1.45(0.24,2.26) | 2.74(1.36,5.50) |
| Elbow deformity | 5 | 1.80(0.24,2.78) | 3.48(1.44,8.42) |
| Hypocalcaemia | 67 | 0.64(0.24,0.93) | 1.56(1.23,1.98) |
| Psoriasis | 459 | 0.39(0.23,0.50) | 1.31(1.19,1.43) |
| Spinal stenosis | 23 | 0.90(0.20,1.39) | 1.87(1.24,2.82) |
| Tenosynovitis | 15 | 1.04(0.17,1.64) | 2.06(1.24,3.42) |
| Panniculitis | 14 | 1.07(0.17,1.69) | 2.10(1.24,3.55) |
| Pain in extremity | 857 | 0.24(0.13,0.32) | 1.18(1.10,1.26) |
| Product use in unapproved indication | 838 | 0.22(0.11,0.30) | 1.16(1.09,1.25) |
| Non-Hodgkin's lymphoma | 24 | 0.78(0.10,1.26) | 1.72(1.15,2.57) |
| Paraesthesia | 428 | 0.22(0.06,0.34) | 1.16(1.06,1.28) |
| Crohn's disease | 200 | 0.29(0.06,0.46) | 1.23(1.07,1.41) |
| Gait disturbance | 549 | 0.20(0.06,0.30) | 1.15(1.05,1.25) |
| Oedema peripheral | 254 | 0.26(0.05,0.41) | 1.20(1.06,1.36) |
| Inflammatory marker decreased | 3 | 2.12(0.05,3.33) | 4.35(1.37,13.78) |
| Gastrointestinal infection | 34 | 0.60(0.03,1.01) | 1.52(1.08,2.13) |
| Bone deformity | 6 | 1.44(0.03,2.35) | 2.72(1.21,6.08) |
| Adverse event | 267 | 0.22(0.02,0.36) | 1.16(1.03,1.31) |
| Inner ear inflammation | 3 | 2.07(0.00,3.28) | 4.21(1.33,13.31) |
| Depression | 543 | 0.14(0.00,0.24) | 1.10(1.01,1.20) |


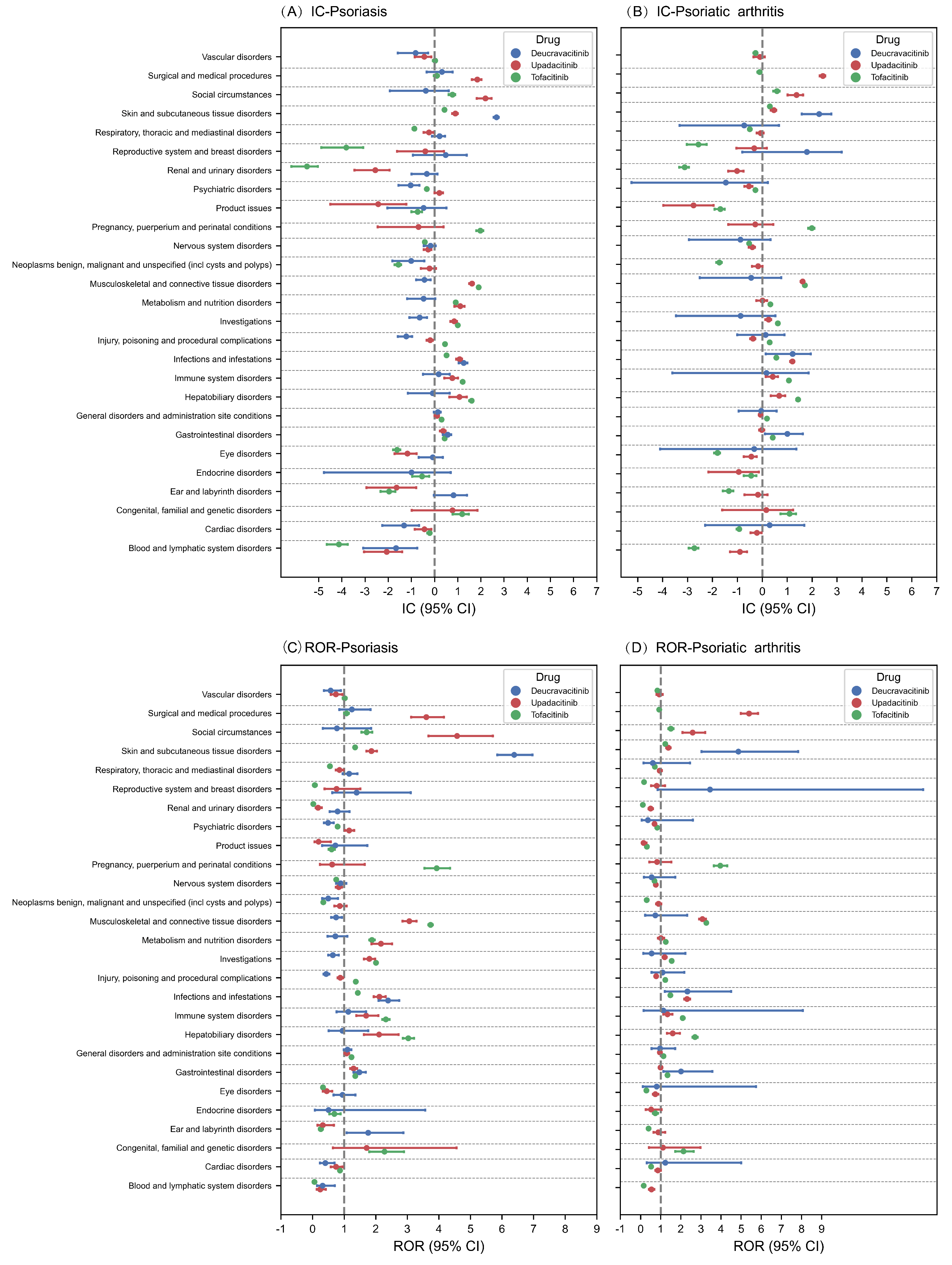


Figure S2 Comparison of signal detection results at the SMQ level between patients with psoriasis and psoriatic arthritis
